# The development of the PET@home toolkit: An experience-based co-design method study

**DOI:** 10.1016/j.ijnsa.2024.100189

**Published:** 2024-03-06

**Authors:** Peter W.A. Reniers, Karin Hediger, Ine J.N. Declercq, Marie-José Enders-Slegers, Roeslan Leontjevas, Debby L. Gerritsen

**Affiliations:** a Faculty of Psychology, Open University of the Netherlands, Heerlen, Netherlands; b Faculty of Psychology, University of Basel, Basel, Switzerland; c Radboud University Medical Center, Radboud Institute for Health Sciences, Radboudumc Alzheimer Centre, Department of Primary and Community Care, Nijmegen, Netherlands

**Keywords:** Community care, Experience-based co-design, Home care, Long-term care, Person-Centred Care, Pets, Toolkit

## Abstract

**Objective:**

The relevance of pets in long-term home care is increasingly recognised because of their effects on health outcomes in clients and the rising number of clients receiving long-term care at home (further referred to as clients receiving home care). Currently, there is a lack of supportive materials that address pet-related challenges within home care. This study aimed to develop a toolkit for clients receiving home care with pets, their family, and professional caregivers using a participatory research approach.

**Methods:**

We used the Experience-Based Co-Design method involving clients receiving home care, family caregivers, and professional caregivers to create tools that are based on both theory and practice. This approach consists of four phases: 1) Exploring topics of emotional significance requiring attention (i.e., key moments) from the perspective of clients receiving home care, family caregivers, and professional caregivers; 2) Collaboratively prioritising these topics, through prioritisation meetings; 3) Developing and refining Toolkit materials through a co-design process; and 4) Evaluating the quality and feasibility of these materials.

**Results:**

Based on the results of a previously-conducted systematic review and individual interviews, we developed a preliminary information booklet and conversation cards. Subsequently, we conducted a total of 28 semi-structured interviews and seven focus groups, including one with representatives of animal interest organisations, such as veterinarians. This process led to the PET@home Toolkit which includes various materials to support pet ownership in home care settings, such as leaflets with advice on communication and animal welfare and an infographic.

**Conclusion:**

The PET@home Toolkit can support professional caregivers and their pet-owning clients receiving home care, family caregivers, and their pets. It may be a valuable addition to providing person-centred care in long-term care at home for clients with pets. The PET@home Toolkit and future updates will be readily available and free to download from May 2024 via the University Knowledge Network for Older Adult Care Nijmegen (www.ukonnetwerk.nl).

**Tweetable abstract:**

The PET@home Toolkit: Supporting pet ownership in long-term care at home.

**What is already known about the topic**

• Pets can be beneficial for clients receiving long-term care at home, but specific challenges exist within the home care setting concerning pets.
• Paying attention to clients’ pets has the potential to mitigate challenges, but currently there are no supportive materials available regarding pets in the home care setting.

Alt-text: Unlabelled box

**What this paper adds**

• This paper introduces the PET@home Toolkit, designed to support clients receiving home care with pets.
• Mitigating challenges related to pets in home care relies on a collaborative effort by clients receiving home care, family caregivers, and professional caregivers.
• Preventing pet-related challenges can foster enduring relationships between clients receiving home care and pets, potentially improving the quality of life for everyone involved, including clients, family caregivers, professional caregivers, and pets.

Alt-text: Unlabelled box

## 1 Background

The global population is ageing, with significant implications for the healthcare system (Abbing, 2016). Consequently, numerous nations are shifting from an intramural care model (i.e., care provided in a residential facility) to an extramural care model, focusing on the concept of ageing-in-place (Abbing, 2016). This transition involves an increasing number of individuals receiving long-term care in their own homes (further referred to as clients receiving home care). Professional caregivers operating in home care will inevitably encounter clients who are also pet owners. While the prevalence of pet ownership tends to decrease with age and disability (Friedmann et al., 2020), the absolute number of pets in home care settings is expected to rise due to the increase in people receiving long-term care at home.

Pet ownership can provide purpose in the lives of clients receiving home care and play vital roles for clients, such as serving as companions, sources of emotional support, and subjects of care (Reniers et al., 2023b; Taeckens et al., 2023). However, the health status of clients receiving home care introduces challenges related to pet care. These challenges encompass the deterioration of the health of clients, reliance on others (e.g., family caregivers) for pet care, potential delays in seeking healthcare (e.g., postponing hospitalisation), and the likelihood of professional caregivers harbouring fears or allergies related to pets (Enders-Slegers and Hediger, 2019; Reniers et al., 2023b). Consequently, while pet ownership by clients receiving home care may have various advantages, it also has potential negative implications within the home care environment.

Offering support regarding pet ownership through healthcare organisations could potentially mitigate burdens within the caregiving system, thereby facilitating the advantages of pet ownership while enriching the quality of life and care for clients receiving home care, family caregivers, and their pets (Rauktis and Hoy-Gerlach, 2020). Attention to pets by professional caregivers, including nurses, case managers, and social workers can help improving support to family caregivers who often play a pivotal role in assisting their loved ones (Law et al., 2021). Moreover, incorporation of issues regarding pets in healthcare can be perceived as a form of person-centred care, as it engages clients receiving home care and family caregivers in the decision-making process concerning their pets, thereby enabling them to explore and express their individual needs (Berglund et al., 2019). Person-centred care has the potential to foster autonomy among clients receiving home care (Jacobs, 2019).

Currently, there is a lack of tools to support clients receiving home care, their family, and professional caregivers regarding issues involving pets. Therefore, the aim of this study was to identify and develop toolkit components for clients, family caregivers, and professional caregivers, aiming to support pet ownership in long-term home care. The purpose of the PET@home Toolkit is to facilitate responsible and enduring relationships between clients receiving home care and their pets. Furthermore, it is aimed to contribute to reducing the possible burden for all parties involved.

## 2 Methods

### 2.1 Research design

We developed the PET@home Toolkit using the Experience-Based Co-Design method (Melchior et al., 2016). In preparation for this study, we conducted a qualitative systematic review (Reniers et al., 2023a) followed by semi-structured interviews using the Consensual Qualitative Research method and administered an online questionnaire to assess the content validity of the outcomes from the interview analyses (Reniers et al., 2023b). The outcomes of both these phases are reported in separate publications (Reniers et al., 2023a; Reniers et al., 2023b), and provided the initial foundation for developing toolkit materials centred around seven themes identified in the literature review, namely relational aspects, reflection and meaning, emotional aspects, aspects of caregiving, physical health, social aspects, and bidirectional behaviour (Reniers et al., 2023a).

This publication focuses on the subsequently conducted Experience-Based Co-Design method, a participatory research approach (Melchior et al., 2016). This approach involves representatives of the target groups under research as co-researchers. Moreover, it aids in creating tools grounded in both science and practice by considering the perspectives and experiences of stakeholders during the development process (Bergold and Thomas, 2012; Melchior et al., 2016). This study was conducted between August 2022 and May 2023 and consisted of four steps.

1. We identified topics that held a special emotional significance for the participants (i.e., key moments) using semi-structured interviews and a focus group.
2. We collaboratively prioritised these key moments (in a prioritisation focus group).
3. We developed and refined toolkit materials through co-design meetings, and
4. We evaluated the quality and feasibility of these materials using semi-structured interviews (Melchior et al., 2016).

**Fig. 1:**
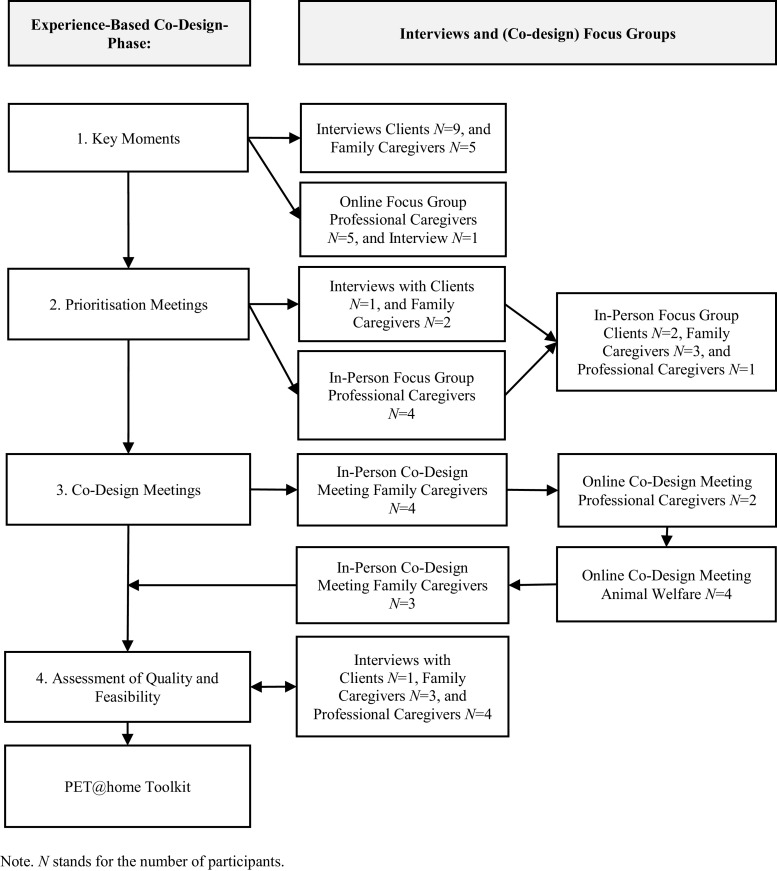
The experience-based co-design process.

See Fig. 1 for the entire Experience-Based Co-Design process and Table 1 for participant’ characteristics.

**Table 1:** Participant characteristics.

Experience-Based Co-Design-Phase:	Method	Participants	Sex	Age Range	Level of Education*	Pet
1. Key Moments	Interviews *N* = 10	Clients *N* = 9; Family Caregivers *N* = 5	Clients: Male *N* = 4, Female *N* = 5; Family Caregivers: Male *N* = 2, Female *N* = 3	Clients: 48–81; Family Caregivers: 50–74	Clients: SE=5 HE=2 Uni=1; Family Caregivers: SE=1 HE=4	Dog *N* = 9; Rabbit *N* = 1
	Focus Group and Interview	Professional Caregivers *N* = 5; Professional Caregiver *N* = 1	Female *N* = 5; Female *N* = 1	23–64; 55	SE=3 HE=2; Uni=1	N/A
2. Prioritisation Meetings	Interviews *N* = 2	Client *N* = 1; Family Caregivers *N* = 2	Client: Male *N* = 1; Family Caregivers: Female *N* = 2	Client: 74; Family Caregivers: 72–74	Client: SE=1; Family Caregivers: SE=1 HE=1	Dog *N* = 2
	Focus Group	Professional Caregivers *N* = 4	Female *N* = 4	43–57	SE=1 HE=2 Uni=1	N/A
	Focus Group	Clients *N* = 2; Family Caregivers *N* = 3; Professional Caregiver *N* = 1	Clients: Male *N* = 1, Female *N* = 1; Family Caregivers: Male *N* = 1, Female *N* = 2; Professional Caregiver: Female *N* = 1	Clients: 74–78; Family Caregivers: 50–72; Professional Caregiver: 43	Clients: SE=2; Family Caregivers: SE=1 HE=1 Uni=1; Professional Caregiver: Uni=1	Dog *N* = 3
3. Co-Design Meetings	Meeting 1	Family Caregivers *N* = 4	Male *N* = 2; Female *N* = 2	50–70	HE=2 Uni=2	Dog *N* = 3; Rabbit *N* = 1
	Meeting 2	Professional Caregivers *N* = 2	Female *N* = 2	55–57	SE=1 HE=1	N/A
	Meeting 3	Animal Welfare Representatives *N* = 4	Female *N* = 4	N/A	N/A	N/A
	Meeting 4	Family Caregivers *N* = 3	Female *N* = 3	50–72	SE=1 HE=1 Uni=1	Dog *N* = 3
4. Quality and Feasibility	Interviews *N* = 7	Client *N* = 1; Family Caregivers *N* = 3; Professional Caregivers *N* = 4	Client: Female *N* = 1; Family Caregivers: Female *N* = 3; Professional Caregivers: Female *N* = 4	Client: 54; Family Caregivers: 54–62; Professional Caregivers 28–62	Client: HP=1; Family Caregivers: SE=1 HE=2; Professional Caregivers: SE=1 HE=3	Dog *N* = 3; Cat *N* = 1

Note. 1. The *N* represents the number of participants or total number of interviews. 2. The level of education is presented as SE/HE/Uni; Secondary Education/Higher Education/University*.*

### 2.2 Research group

The research group included two PhD students, one specialising in human-animal research and the other in geriatric care research. Additionally, the group consisted of two experts in human-animal research and two experts in geriatric care research. The experts possess extensive experience in conducting a diverse range of projects involving various forms of qualitative research.

During research group discussions, we ensured opportunities for reflexivity. Notably, one of the researchers herself had experience as a family caregiver for a client receiving home care who owned a pet.

### 2.3 Participants and recruitment

The project involved clients receiving home care, family caregivers, and professional caregivers throughout its various phases. Participants could participate in multiple phases of the project and were recruited through two community-care organisations and an organisation dedicated to supporting family caregivers. The community-care organisations directly distributed our information letter to clients receiving home care who owned pets.

To incentivise participation, those who took part in the study during their leisure time received a 20-euro gift voucher. Employees from one of the community-care organisations could participate in the study during their regular working hours.

### 2.4 Procedures

In-person individual and duo-interviews and online focus groups were recorded using either a digital voice recorder or the recording feature in the secure Microsoft Teams environment of the Open Universiteit. Recordings were transcribed using a selective transcription approach, where the text was edited to include only relevant information based on the aim of the study (Sandelowski, 1994). Resulting transcriptions were checked by a second researcher for clarity, relevance, and completeness (two research group members along with several internship students). During co-design meetings, two or three research group members who were present took comprehensive notes to record relevant information.

Furthermore, we enriched the toolkit through collaboration with subject-matter experts (such as The Dutch Society for the Protection of Animals and a veterinarian). Additionally, a graphical designer and communication expert provided input on the layout of materials, and the clarity and readability of the text.

### 2.5 Phase 1: key moments

We conducted semi-structured interviews to explore the experiences of clients receiving home care and family caregivers with pets. Subsequently, we organised an online focus group and an individual interview with professional caregivers to explore their experiences with pets (see Fig. 1). During interviews with clients receiving home care and family members, we asked questions like, ‘*Can you share an example of a particular moment [related to your pet] that had either a positive or negative emotional impact on you?*’ In the focus group with professional caregivers, we used prompts such as, *‘Can someone provide an example of a specific challenge related to one of your clients’ pets that you have personally experienced?’.* Two researchers independently analysed the data collected from the interviews and focus groups using tabular analysis (Robinson, 2022), followed by a discussion until consensus was reached on the outcomes.

### 2.6 Phase 2: prioritisation meetings

We assessed the key moments for their relevance and importance through in-person meetings. The first author conducted an interview with a family caregiver and a duo-interview with a client and a family caregiver in their own homes. Additionally, we utilised an in-person focus group with professional caregivers (See Fig. 1).

We presented the key moments by sharing statements related to each key moment and playing audio excerpts from interviews and focus group discussions to the participants. Participants assessed the relevance and importance of these key moments by scoring them on a provided form. In the focus group, the professional caregivers collectively determined the most relevant and important key moments.

Subsequently, we organised a mixed group of clients receiving home care, family caregivers, and professional caregivers (see Fig. 1) to determine which topics had the highest priority for the toolkit and how to effectively address them.

### 2.7 Phase 3: co-design meetings

We held four sessions consisting of in-person and online groups with clients receiving home care, family caregivers, caregiving professionals, and a meeting with representatives from animal welfare organisations (see Fig. 1).

The sessions followed an iterative approach, resembling the Plan-Do-Check-Act model, which is a circular model promoting continuous improvement of materials and procedures (Ramos et al., 2022). Consensus was reached through group discussions, minimising power differences among group members. A strategy used to achieve this was allowing informal conversations with refreshments before the meetings started, helping participants feel comfortable with the setting and with each other. This approach facilitated open discussions, with all participants sharing their input (Bergold and Thomas, 2012; Eckstein, 2016; Melchior et al., 2016; Sirimsi et al., 2023). The toolkit materials were refined after each session.

### 2.8 Phase 4: assessment of quality and feasibility

Finally, we conducted an assessment of the quality and feasibility of the materials (Melchior et al., 2016). This assessment included interviews aimed at evaluating the intended comprehension of texts (Willis, 1999). The interviews involved a client receiving home care, family caregivers, and professional caregivers (see Fig. 1) who had not participated in the co-design meetings. An example question asked during these interviews was *‘How do you perceive the ease of use of the toolkit? If you find it challenging, what suggestions do you have to make it simpler?’.* The final iteration of toolkit materials occurred after this phase.

### 2.9 Ethical procedures

All participants provided informed consent, which they could provide in writing or through an audio or video recording. We stored the informed consent separately from the interview transcripts, on a secure university drive. After transcription and analyses, we deleted audio and video recordings of the interviews to secure privacy of participants. This procedure adhered to the study protocol approved by the Open Universiteit's ethics committee (U202206075).

## 3 Results

### 3.1 Phase 1 and 2: key moments and prioritisation meetings

The research group initially identified 14 key moments from clients receiving home care and family caregivers and 17 key moments from professional caregivers (Supplementary Material Table A1). During the prioritisation meetings clients receiving home care and family caregivers regarded five key moments as most relevant and important to them. In a focus group professional caregivers considered six additional key moments as most relevant and important to them (see Table 2).

**Table 2:** Most relevant and important key moments.

	Clients and Family Caregivers		Professional Caregivers
1.	Saying goodbye to a pet can be challenging	1.	Clients who forget to provide care for their pets.
2.	Pet ownership may lead to postponing healthcare or transitioning to a nursing home.	2.	Pet ownership may lead to postponing healthcare or transitioning to a nursing home.
3.	The absence of a family caregiver can create challenges in caring for pets.	3.	Pets can form a bond between clients and professional caregivers.
4.	Healthcare organisations consider the fear and allergies of staff towards pets.	4.	The additional burden on family caregivers may result in less attention to pet care.
5.	Some clients believe they can adequately care for their pets and may not appreciate interference from professional caregivers.	5.	There are no existing agreements regarding a client’ pet.
		6.	Cases of animal abuse by clients.

Note. These key moments were indicated as the most relevant or important during the initial prioritisation meetings.

The key moments presented in Table 2 were discussed in the following in-person prioritisation focus group, comprising clients receiving home care, family caregivers, and professional caregivers (Fig. 1). By this, consensus was reached about priority topics that needed to be addressed in the toolkit, namely:

1. Promoting understanding of the roles that pets play for clients receiving home care.
2. Creating awareness of potential pet-related challenges and their impact on caregiving relationships.
3. Encouraging discussions about pet-related issues and facilitating agreements concerning pet care within the clients’ social network.
4. Emphasising the importance of animal wellbeing.

### 3.2 Phase 3: co-design meetings

Throughout the co-design meetings, participants developed various toolkit materials. For a summary of the materials created for each priority topic, see Table 3. Additionally, Table 4 provides an overview of the aims, target groups, methods of utilisation, and indicators of successful implementation of these materials. Toolkit materials in Dutch are available as supplementary files.

**Table 3:** PET@home Toolkit materials related to the priority topics.

	Promoting understanding of the roles pet play for clients	Creating awareness of pet-related problems and their impact on caregiving relationships	Encouraging discussions about pet-related issues and facilitating agreements	Emphasising the importance of animal wellbeing
All stakeholders	Infographic			
Organisations	Leaflet with practical advice			
Professional caregivers	Conversation cards	Checklist care plan talks	Conversation cards; Leaflet communication; Checklist care plan talks	Leaflet animal welfare
Clients and family caregivers	Information booklet			

Note. Some materials were intended to support various priority topics.

**Table 4:** PET@home toolkit specification of materials.

Material	Aims	Target group	How to use	Indicators of successful implementation
Information booklet	1. Informing about the roles of pets 2. Fostering clients’ autonomy 3. Fostering mutual understanding 4. Supporting in communication and decision-making	Clients and family caregivers	1. Professional caregivers provide the booklet to clients 2. Clients and family discuss the pet 3. Clients and family make agreements regarding the pet	1. Clients read the booklet 2. Agreements have been made
Checklist care plan talks	1. Assessing concerns related to pet care	Professional caregivers	1. Caregivers discuss the concerns during a care plan talk 2. Caregivers regularly evaluate agreements	1. The checklist is discussed regularly 2. Concerns are registered in the (digital) care plan
Leaflet communication	1. Providing tips to talk about the pet	Professional caregivers	1. Caregivers read the leaflet	1. The tips are used
Leaflet animal welfare	1. Informing about animal welfare	Professional caregivers	1. Caregivers read the leaflet	1. Concerns related to pet welfare are recognised 2. During emergencies, caregivers know where to go
Conversation cards	1. Informing about the roles of pets 2. Providing tips for conversation topics 3. Fostering mutual understanding	Professional caregivers	1. Caregivers use the cards	1. Pets are discussed with clients 2. Pets are discussed with colleagues
Infographic	1. Explaining the toolkit	All stakeholders	1. The infographic is visibly placed on a wall in the organisation 2. The infographic is shared digitally with stakeholders	1. Stakeholders are aware of the toolkit and its contents
Leaflet with practical advice	1. Providing practical organisational advice	Healthcare organisations	1. The advice is implemented in the organisation	1. Practical advice is implemented in the organisation

### 3.3 Information booklet

Participants emphasised the need to include all relevant materials for clients receiving home care and family caregivers in a single comprehensive information booklet. The booklet covers a wide range of topics related to all four priority topics, such as the significance of pets for clients receiving home care, potential challenges, available support options for pet care (e.g., volunteer services), means of environmental enrichment for animals, communication tips (e.g., employing open-ended questions), and an agreement template (e.g., they can register addresses for temporary or permanent pet care).

### 3.4 Checklist care plan talks

During the meetings, participants proposed that professional caregivers required guidance during care plan discussions. The checklist includes factors that the client may experience, such as cognitive, physical, or social issues that may complicate pet care.

### 3.5 Leaflet communication

The leaflet for professional caregivers provides practical guidance on including pets in daily conversations with clients receiving home care. Participants indicated that it would be desirable for professional caregivers to talk about pets with their clients to improve caregiving relationships and manage clients’ expectations concerning pets, such as that professional caregivers will not provide care for pets.

### 3.6 Leaflet animal welfare

Participants suggested that professional caregivers need support concerning animal welfare. Therefore, the leaflet provides information to help professional caregivers to quickly understand animal well-being indicators and provides contact information for an emergency.

### 3.7 Conversation cards

The systematic literature review (Reniers et al., 2023a) and subsequently conducted Consensual Qualitative Research method study (Reniers et al., 2023b) led to the development of seven conversation cards for professional caregivers. Each card explains a distinct theme; for example the bond between a client receiving home care and their pet. Moreover, these cards promote mutual understanding by presenting perspectives on a particular theme from different stakeholders.

### 3.8 Infographic

This product for all stakeholders takes the form of a poster. The poster conveys information about the toolkit including its aims, materials, and target groups. Its purpose is to inform professional caregivers, clients receiving home care, and family caregivers, about the toolkit and its intended use.

### 3.9 Leaflet practical advice

During the sessions participants provided practical advice that healthcare organisations could implement in their policies and procedures to effectively include clients’ pets into the caregiving practice. Examples of such advice included developing an organisational vision regarding pets of clients receiving home care and appointing a designated person within the organisation to handle pet-related ethical matters.

### 3.10 Phase 4: quality and feasibility evaluation and cognitive interviews

All participants expected that the toolkit would have benefits for their daily lives. For example, they expressed that the conversation cards would be useful for educating stakeholders, and they considered that the agreement template in the booklet could be helpful for documenting agreements about pet care with others. Overall, participants indicated that the toolkit raised their awareness about the significance of pets for clients receiving home care, the potential challenges that could arise over time, and support options.

Participants provided suggestions for improvement, such as including more writing space in the agreement template and adding a local guide for pet services.

During the interviews, participants suggested further simplification of the texts. For instance, they recommended using ‘having a conversation’ instead of the term ‘communication’ and ‘a pleasant environment for pets’ rather than ‘environmental enrichment’.

## 4 Discussion

Currently, there is a lack of supportive materials for pet ownership in the home care setting. This study aimed to identify and develop toolkit components that supports pet ownership for long-term care clients receiving home care, family caregivers, and professional caregivers. The PET@home Toolkit provides tools to promote mutual understanding, raise awareness of potential challenges and support options, and facilitate conversations, decision-making, and planning. Ultimately, it aims to improve outcomes of pet ownership within home care settings, enhancing caregiving relationships, reducing caregiver burden, and promoting animal welfare.

Professional caregivers can play a vital role in facilitating conversations about pets and offering practical advice. A Swedish study found that professional caregivers, clients receiving home care, and family caregivers all play a role in risk prevention in clients’ own homes (Lekman et al., 2023). Sharing information, fostering caregiving relationships, and collaborating are vital for achieving common goals (Lekman et al., 2023). It can be argued that mitigating challenges related to pet ownership in home care requires a similar approach. However, the authors of the Swedish study also noted certain challenges regarding risk prevention that may also apply when assessing support requirements concerning pets. For instance, differing perspectives among stakeholders, clients’ resistance to changing risky habits, and uncooperative family members (Lekman et al., 2023). When professional caregivers encounter these challenges, it may complicate effective use of the toolkit.

Utilising the PET@home Toolkit effectively will rely on a collaborative effort by professional caregivers, clients receiving home care, and their families, who are best suited to assess, determine, and arrange support needs concerning their pets. The toolkit materials, including the information booklet, leaflet on communication, and checklist for care plan talks, all emphasise clear and open communication among all stakeholders in the caregiving triad. This can lead to an exchange of information, foster caregiving relationships, and build trust among stakeholders positively impacting the self-management abilities of clients receiving home care (Doekhie et al., 2019). Using the toolkit aligns with the principles of person-centred care, which involves engaging clients in decision-making, addressing their needs, promoting continuity and predictability, and contributing to their overall well-being (Kristensen et al., 2017; Sundler et al., 2020). While person-centred care has gained attention, further research and tool development is needed to support professional caregivers in engaging clients in their care in a supportive, explorative, and respectful manner (Sundler et al., 2020). The positive evaluation of the toolkit's quality and applicability indicates its potential to improve the quality of life for all involved.

## 5 Limitations, strengths, and future research

A limitation of the toolkit is that we did not yet test the toolkit materials in practical home care settings. Instead, participants based their opinions on their personal experiences.

A second limitation is that home care clients were underrepresented, and some group discussions had lower participation levels than intended (e.g., due to participant cancellations often due to illness). However, despite these smaller groups, we were able to conduct in-depth and detailed discussions on various topics.

A final limitation may be that combining key moments for clients and family caregivers introduces bias. Their experiences of the human-animal bond may differ significantly.

One notable strength of this project lies in the collaborative involvement of clients receiving home care, family caregivers, professional caregivers, and experts in developing the toolkit materials. All participants had relevant experience regarding pets within the home care context.

A second strength is the user-friendliness of the toolkit. It presents potential challenges and offers a variety of support options, facilitating discussions among stakeholders to customise the needed pet care support based on their individual circumstances.

A last strength is that we extended the original Experience-Based Co-Design method with a preparatory literature review and a study utilising the Consensual Qualitative Research method that involved stakeholders as well. This helped to gather a wealth of information concerning pet ownership in long-term care at home before conducting the Experience-Based Co-Design method.

Future research should evaluate the toolkit materials in real-life situations, such as during care plan discussions, while collecting feedback and assessments from users. Additionally, an application study could determine the effect of toolkit implementation on various outcome measures, such as mood symptoms in clients receiving home care and family caregivers, the severity of caregiver burden, and client and family caregivers’ quality of life.

To ensure its applicability in other countries, gathering input from stakeholders in those regions is essential, potentially through methods like focus groups. This should be relatively simple to execute, and the input would facilitate the adaptation of the toolkit to home care settings in other countries.

## 6 Conclusions

We developed a user-friendly toolkit using a participatory research approach to support clients receiving long-term care at home, family caregivers, and professional caregivers regarding pets. This is a valuable contribution for improving long-term care at home due to the current lack of available tools concerning pet ownership. Increasing numbers of people are receiving long-term home care and, consequently, more clients live with pets. Moreover, pets are important to their owners, and pets can have positive effects on various outcomes, such as reducing depression, increasing social connections, and providing purpose to clients’ lives. The toolkit has the potential to improve the quality of life for clients receiving home care with pets, family caregivers, professional caregivers, and pets alike.

## Data availability

Participants were assured raw data would remain confidential and would not be shared.

## CRediT authorship contribution statement

**Peter W.A. Reniers:** Formal analysis, Investigation, Writing – original draft, Project administration. **Karin Hediger:** Conceptualization, Methodology, Writing – review & editing, Supervision. **Ine J.N. Declercq:** Formal analysis, Investigation, Writing – review & editing. **Marie-José Enders-Slegers:** Conceptualization, Methodology, Writing – review & editing, Supervision, Funding acquisition. **Roeslan Leontjevas:** Conceptualization, Methodology, Writing – review & editing, Supervision, Funding acquisition. **Debby L. Gerritsen:** Conceptualization, Methodology, Writing – review & editing, Supervision, Funding acquisition.

## Declaration of competing interest

None.

## Appendix Supplementary materials

Image, application 1

Image, application 2

Image, application 3

Image, application 4

Image, application 5

Image, application 6

Image, application 7

Image, application 8
