# Supplementary material for: The development of the PET@home toolkit: An experience-based co-design method study
Source: Int J Nurs Stud Adv. 2024 Mar 6;6:100189. doi: 10.1016/j.ijnsa.2024.100189 (PMC11080344; doi:10.1016/j.ijnsa.2024.100189)
Supplement: Supplementary file 1 [file mmc1.docx]

Table A1. Key Moments after Phase 1

|  | **Clients and family caregivers** |  | **Professional caregivers** |
| --- | --- | --- | --- |
| 1.* | Saying goodbye to a pet can be challenging | 1. | Not everyone has a support network to assist with pet care. |
| 2. | Deciding what to do with a pet when the owner is no longer able to care for them (e.g., due to hospitalisation). | 2.* | Clients who forget to provide care for their pets. |
| 3.* | Pet ownership may lead to postponing healthcare or transitioning to a nursing home. | 3.* | Pet ownership may lead to postponing healthcare or transitioning to a nursing home. |
| 4. | The health deterioration of clients can make pet care increasingly difficult for family caregivers. | 4. | Severe contamination of clients’ residences due to pets. |
| 5.* | The absence of a family caregiver can create challenges in caring for pets. | 5. | The pet can serve as a means of communication with clients (e.g., those with dementia). |
| 6. | Caregivers might encounter difficulties while providing care in the presence of pets. | 6.* | Pets can form a bond between clients and professional caregivers. |
| 7.* | Healthcare organisations consider the fear and allergies of staff towards pets. | 7. | Sometimes, it is necessary for a client to part ways with their pet. |
| 8. | Pet care can lead to discussion and tension between family caregivers and clients. | 8.* | The additional burden on family caregivers may result in less attention to pet care. |
| 9.* | Some clients believe they can adequately care for their pets and may not appreciate interference from professional caregivers. | 9. | At times, the wellbeing of the pet takes precedence over that of the clients. |
| 10. | Clients and family caregivers may expect more support from their network regarding pet care (e.g., from children). | 10.* | There are no existing agreements regarding a client’ pet. |
| 11. | Pets can display aggressive behaviour towards other people. | 11. | Agreements between professional caregivers and clients may gradually become less effective. |
| 12. | Clients appreciate it when others give attention to their pet. | 12. | Different pets have different needs. |
| 13. | Some clients may be unable to perform certain caregiving tasks, such as walking the dog. | 13. | Professional caregivers who fear pets may find it challenging to bond with a client who owns a pet. |
| 14. | Some clients have to come to terms with the fact that they can no longer care for a pet. | 14. | In the presence of pets, some professional caregivers may exceed their limits (e.g., due to anxiety). |
|  |  | 15.* | Cases of animal abuse by clients. |
|  |  | 16. | Some clients expect professional caregivers to also perform caregiving tasks for the pet. |
|  |  | 17. | Clients may become irritated by professional caregivers who interfere with pet care. |

Note. The key moments marked with an asterisk (*) were indicated as the most relevant and/or important issues for discussion during the initial prioritisation meetings. Subsequently, these moments were addressed during the prioritisation group discussion involving clients receiving home care, family caregivers, and professional caregivers.
