# Supplementary material for: The development of the PET@home toolkit: An experience-based co-design method study
Source: Int J Nurs Stud Adv. 2024 Mar 6;6:100189. doi: 10.1016/j.ijnsa.2024.100189 (PMC11080344; doi:10.1016/j.ijnsa.2024.100189)
Supplement: Supplementary file 2 [file mmc2.pdf]

Inventarisatielijst

Checklist aandachtspunten cliënten met huisdieren, te gebruiken tijdens zorgplangesprekken

PET@home
Als het baasje zorg nodig heeft

Eigenaar huisdier: ______________________ **Telefoonnummer:** ______________________
Type huisdier: ______________________ **Geboortedatum huisdier:** ______________________
Ras huisdier: ______________________
Naam huisdier: ______________________
Contactpersoon: ______________________ **Telefoonnummer:** ______________________
Dierenarts huisdier: ______________________ **Telefoonnummer:** ______________________

Neem de PET@home brochure mee naar de cliënt en neem hem samen door. Bespreek dat de verantwoordelijkheid voor het huisdier bij cliënt en diens naasten ligt. Evalueer regelmatig of afspraken nog actueel zijn.

- ☐ De PET@home brochure is verstrekt en doorgenomen
- ☐ De verantwoordelijkheden zijn duidelijk gemaakt

Onderstaande punten zijn opgenomen in het digitale zorgplan:

- ☐ De aanwezigheid van het huisdier
- ☐ Bijzonderheden over het huisdier
- ☐ Contactpersoon (anders dan de cliënt) voor het huisdier

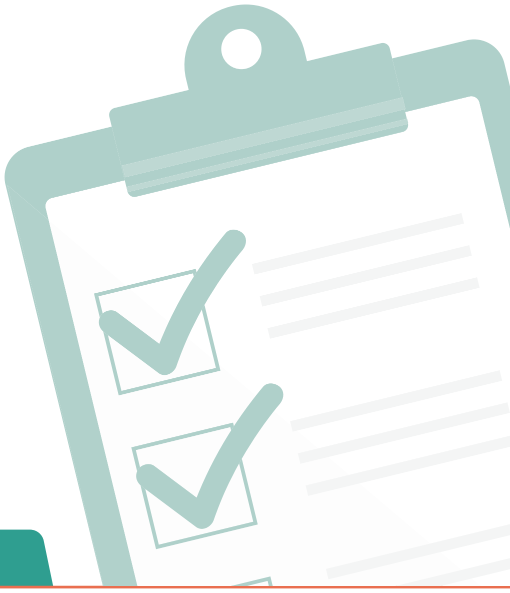

Mede mogelijk gemaakt door:
ZonMw · Open Universiteit · Radboudumc · UKON Universitair Kennisnetwerk Ouderenzorg Nijmegen · de Zorgboog voor alle generaties

Aandachtspunten m.b.t. cliënt

Bij onderstaande punten is het extra belangrijk dat de cliënt en naasten afspraken maken over het huisdier:

Lichamelijk

☐ De cliënt heeft lichamelijke problemen die invloed kunnen hebben op de zorg voor het huisdier. Denk hierbij aan problemen bij het uitlaten, voeren, verschonen of verzorgen.

Eventuele toelichting:

Cognitief

☐ De cliënt heeft cognitieve problemen die van invloed kunnen zijn op de zorg voor het huisdier. Denk hierbij aan verdwalen, het juiste eten geven of op tijd naar de dierenarts gaan.

Eventuele toelichting:

Psychosociaal

☐ De cliënt vertoont een emotionele disbalans die invloed kunnen hebben op de zorg voor het huisdier. Denk hierbij aan ernstige somberheid of manie.

Eventuele toelichting:

☐ De cliënt krijgt geen hulp van naasten (familie, vrienden, kennissen, buren) bij de zorg voor het huisdier. Denk hierbij aan hulp bij uitlaten, voeren, verzorgen, oppassen, dierenartsbezoek.

Eventuele toelichting:

De fysieke omgeving

☐ De fysieke leefomgeving is niet in orde. Denk hierbij aan gebrek aan hygiëne of valgevaar (bv. voederbak in looppad).

Eventuele toelichting:

☐ Extra observaties of mogelijke aandachtspunten.

Eventuele toelichting:

Aandachtspunten m.b.t. huisdier

Bij onderstaande punten is het extra belangrijk dat de cliënt en naasten afspraken maken over het huisdier:

- [ ] Het huisdier lijkt pijn te ervaren of een verwonding of ziekte te hebben.

Eventuele toelichting:

- [ ] Het huisdier lijkt angst en of stress te ervaren. Denk hierbij aan terugdeinzen, trillen, haren of veren uittrekken, of plotseling ander gedrag ten opzichte van eigenaar.

Eventuele toelichting:

- [ ] Het huisdier lijkt onvoldoende ruimte te hebben of wordt onvoldoende uitgelaten.

Eventuele toelichting:

- [ ] Er zijn geen adequate voorzieningen voor het huisdier aanwezig. Denk hierbij aan een mand of kussen om op te liggen, een kattenbak of kooi.

Eventuele toelichting:

- [ ] Er zijn problemen met de interactie tussen het huisdier en cliënt en/of naasten. Denk hierbij aan afwezigheid van affectie of juist de aanwezigheid van agressief of afstandelijk gedrag.

Eventuele toelichting:

- [ ] Er zijn problemen met de interactie tussen het huisdier en zorgverleners. Denk hierbij aan agressief of angstig gedrag.

Eventuele toelichting:

- [ ] Extra observaties of mogelijke aandachtspunten.

Eventuele toelichting:
