# Supplementary material for: The development of the PET@home toolkit: An experience-based co-design method study
Source: Int J Nurs Stud Adv. 2024 Mar 6;6:100189. doi: 10.1016/j.ijnsa.2024.100189 (PMC11080344; doi:10.1016/j.ijnsa.2024.100189)
Supplement: Supplementary file 3 [file mmc3.pdf]

Fysieke gezondheid

Beweging

- Een huisdier stimuleert om meer te bewegen.
- Verzorgen, wandelen of spelen met een huisdier draagt bij aan beweging.

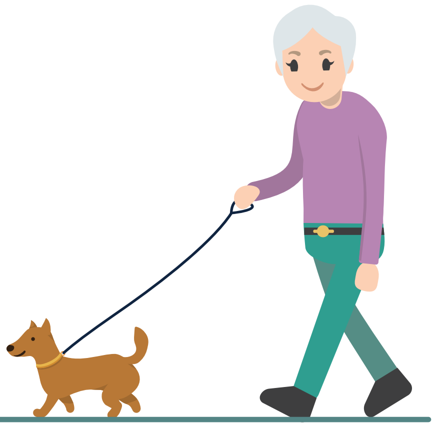

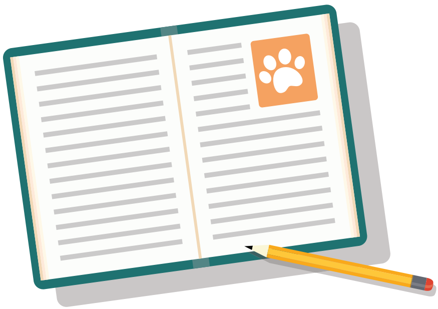

Structuur

- Een huisdier vraagt geregeld aandacht.
- Een huisdier geeft aan wanneer die wil eten of naar buiten wil.
- Een huisdier kan zorgen voor structuur in het dag- en nachtritme van de cliënt.

Ontspanning

- Het aaien, de verzorging, de aanwezigheid en het praten met een huisdier kan ontspanning bieden.
- Het uitlaten van de hond kan ontspanned werken.

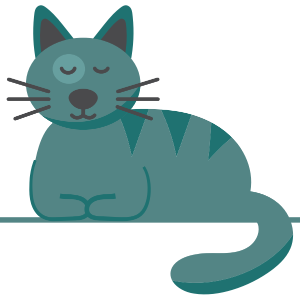

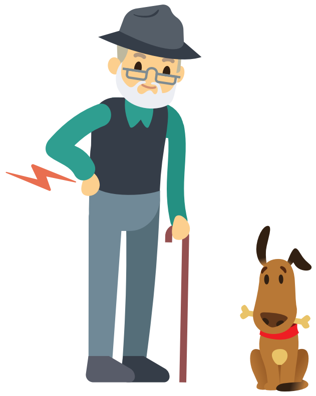

Afleiding

- De aanwezigheid van een huisdier kan afleiden van pijn en ongemak.

Een cliënt vertelt:

‘Het dier is voor mij heel belangrijk, omdat het me tot rust laat komen.’

PET@home
Als het baasje zorg nodig heeft

Cliënten vertellen:

- Ik zet mijn duif op mijn schouder. Dit ontspant mij.
- Het borstelen van mijn konijn is mijn meditatiemoment.
- Wandelen en spelen met mijn huisdier zorgt voor beweging. Ik voel mij fitter.
- Ik moet de hond uitlaten, ook als ik mij niet goed voel.
- Zonder mijn huisdier zou ik niet buitenkomen.

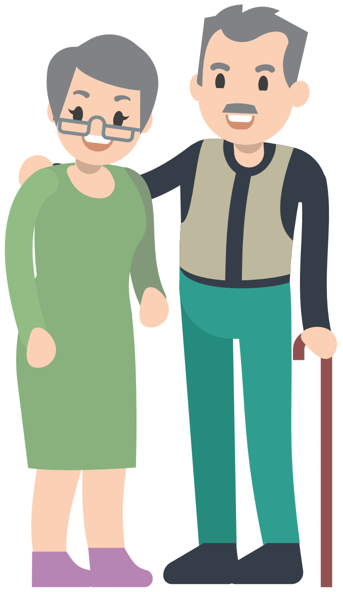

Mantelzorgers vertellen:

- Door te wandelen met de hond voel ik mij fitter en kom ik tot rust.
- Ik laat samen met mijn partner onze hond uit.

Zorgverleners vertellen:

- Met het huisdier stimuleer ik een cliënt om te bewegen.
- Ik houd rekening met de structuur rondom het huisdier en pas mij hierop aan.
- Ik maak mij ongerust wanneer een cliënt niet meer goed voor een huisdier zorgt.
- Een cliënt met dementie kan verdwalen tijdens het uitlaten van de hond. Dit kan gevaarlijk zijn.
- Een huisdier biedt structuur vanwege de zorgtaken. Dit geeft houvast aan cliënten met dementie.

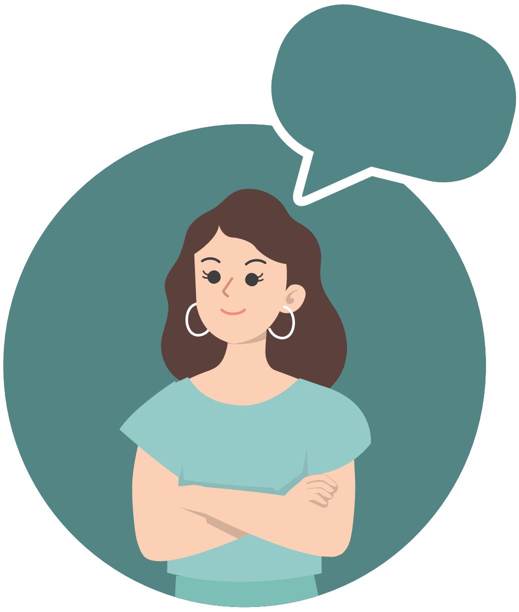

Dit project is mede mogelijk gemaakt door:

ZonMw · Open Universiteit · Radboudumc · UKON Universitair Kennisnetwerk Ouderenzorg Nijmegen · de Zorgboog voor alle generaties

Sociale aspecten

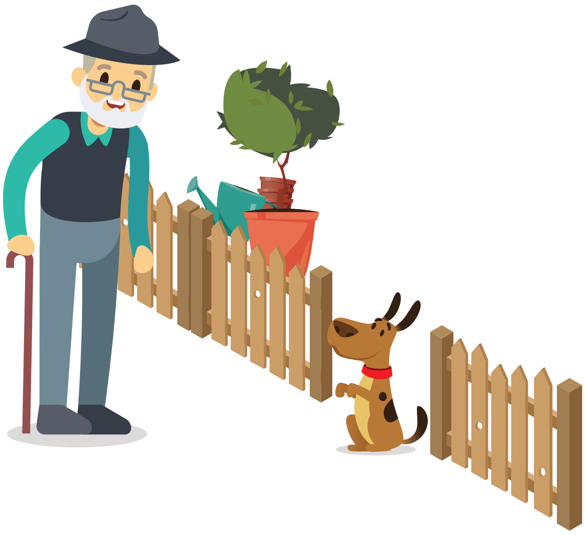

Eenzaamheid

- Cliënten voelen zich minder eenzaam door een huisdier.
- Cliënten met een huisdier komen nooit in een 'leeg' huis. Het huisdier is altijd aanwezig.
- Cliënten voelen zich minder eenzaam door te praten tegen hun huisdier.
- De aanwezigheid van een huisdier zorgt ervoor dat cliënten altijd iets levends om zich heen hebben.

Contact met andere mensen

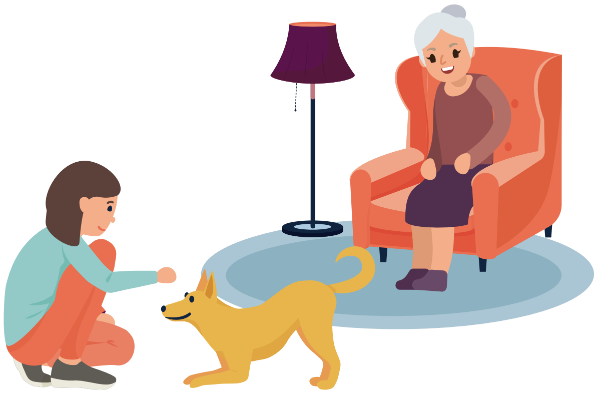

- Een huisdier bevordert het contact met anderen, bijvoorbeeld tijdens het wandelen met de hond.
- Een huisdier werkt als 'ijsbreker' bij visite.
- Een ander neemt zorgtaken voor het huisdier over. Hierdoor ontstaan extra contactmomenten.
- Cliënten kunnen lid worden van bijvoorbeeld een vogelvereniging. Dit zorgt voor extra sociale contacten.
- Een huisdier kan bijdragen aan sociale controle. Buren zien dat de cliënt niet is gaan wandelen met de hond en gaan kijken of er iets aan de hand is.

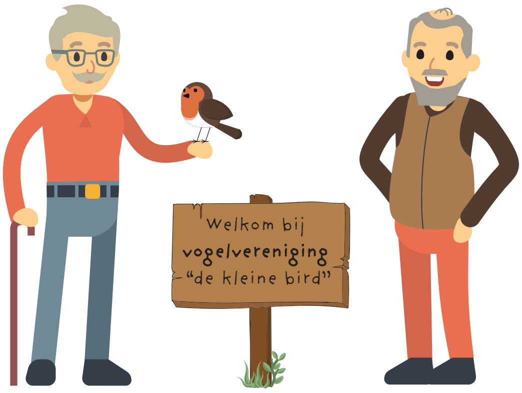

Een cliënt vertelt:

'Of ze weten dat je een kat hebt en vragen dan: hoe is het met de kat?'

PET@home
Als het baasje zorg nodig heeft

Cliënten vertellen:

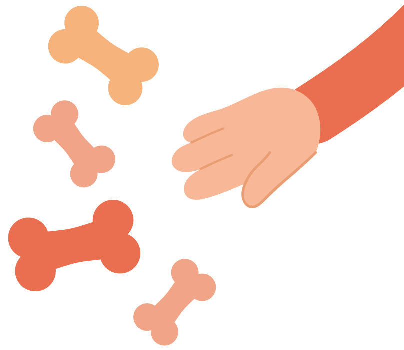

- Ik vind het fijn om mensen tegen te komen tijdens het uitlaten van de hond.
- Ik vertel aan mijn thuiszorghulp wie ik ben tegengekomen tijdens het uitlaten van de hond.
- De buurman geeft mijn huisdier snoepjes en dit bevordert het contact.
- Ik vind het makkelijk om over mijn huisdier te praten.
- Ik praat tegen mijn huisdier en voel mij hierdoor minder eenzaam.

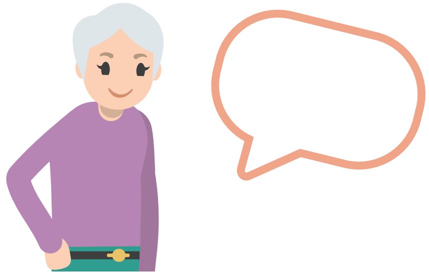

Mantelzorgers vertellen:

- Doordat ik met de hond wandel, heb ik vaker contact met andere mensen.
- Ik praat met mijn partner over ons huisdier.

Zorgverleners vertellen:

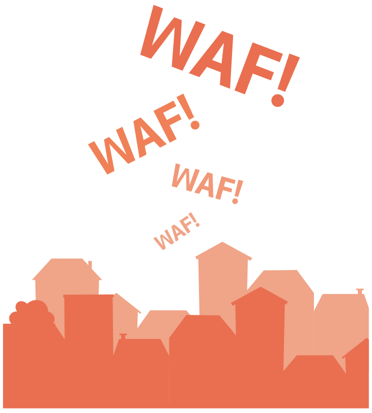

- Ik vind het prettig om te zien dat cliënten met een huisdier minder eenzaam zijn.
- Cliënten die een hond niet meer kunnen uitlaten, hebben ook geen extra sociale contacten.
- Zodra cliënten niet meer goed voor het huisdier zorgen, leidt dit mogelijk tot overlast in de buurt: denk aan blaffen van de hond.

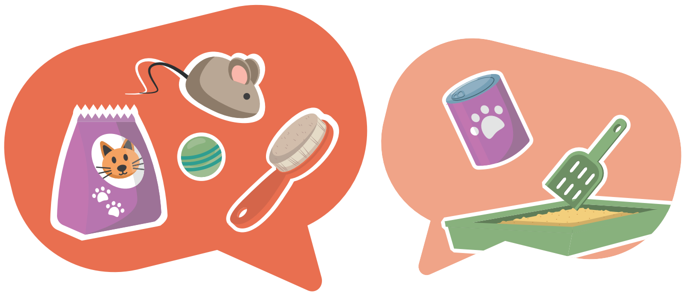

- Ik merk dat een huisdier een fijn gespreksonderwerp is, zeker wanneer ik even niet weet wat te zeggen.
- Ik geef het huisdier aandacht. Hierdoor maak ik ook makkelijker verbinding met de cliënt.

Dit project is mede mogelijk gemaakt door:

ZonMw | Open Universiteit | Radboudumc | UKON Universitair Kennisnetwerk Ouderenzorg Nijmegen | de Zorgboog voor alle generaties

Interactie tussen baasje en huisdier

Contact met het huisdier

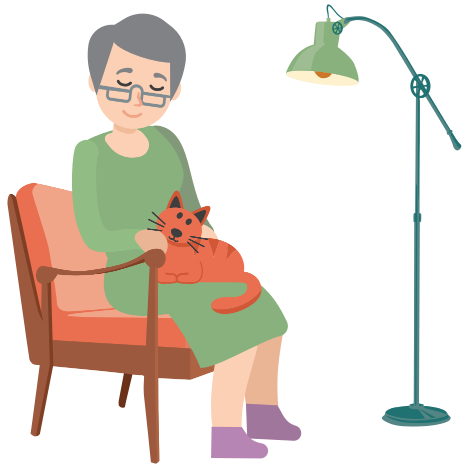

- Een huisdier vraagt aandacht van het baasje.
- Cliënten vinden het fijn om hun huisdier te aaien en te knuffelen. Iets wat huisdieren ook prettig lijken te vinden.
- Cliënten ervaren een rustgevend gevoel door lichamelijk contact met hun huisdier.
- Cliënten praten tegen hun huisdier.

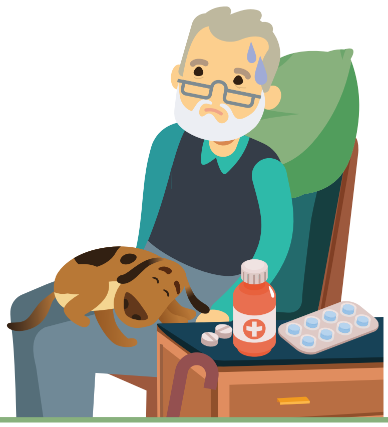

Nabijheid

- Huisdieren en hun baasje zoeken vaak elkaars nabijheid op.
- Cliënten voelen zich geliefd en belangrijk wanneer hun huisdier de nabijheid zoekt.
- Een huisdier wijkt niet van de zijde van het baasje, ook als het baasje ziek is.

Gedrag

- Huisdieren voelen veranderingen aan in hun omgeving.
- Huisdieren voelen hun baasje aan en reageren op het gedrag van hun baasje.
- Cliënten hebben vaak het idee dat hun huisdier hen begrijpt.

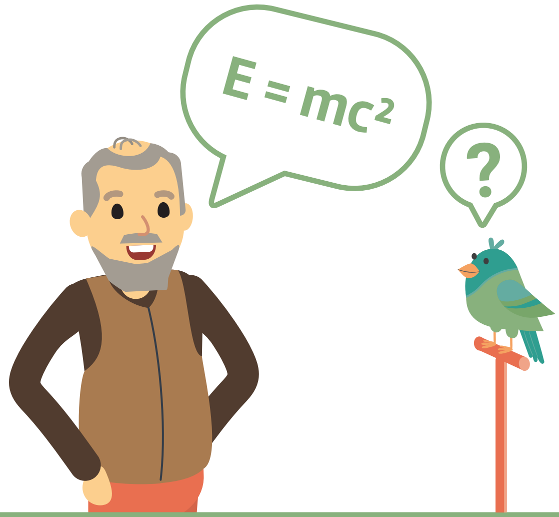

Een mantelzorger vertelt:

'Als je hier op de bank ligt, dan komt ze altijd naar je toe.'

PET@home
Als het baasje zorg nodig heeft

Cliënten vertellen:

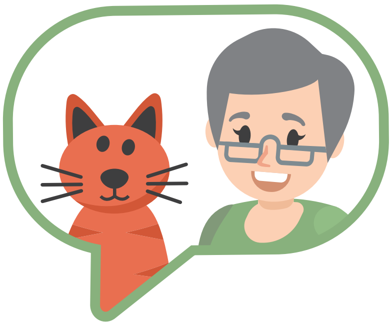

- Ik praat tegen mijn huisdier.
- Ik vind het fijn als mijn huisdier bij mij komt en aandacht vraagt.
- Ik knuffel graag met mijn huisdier.
- Ik neem mijn huisdier overal mee naartoe.
- Ik voel me prettig als mijn huisdier in de buurt is.

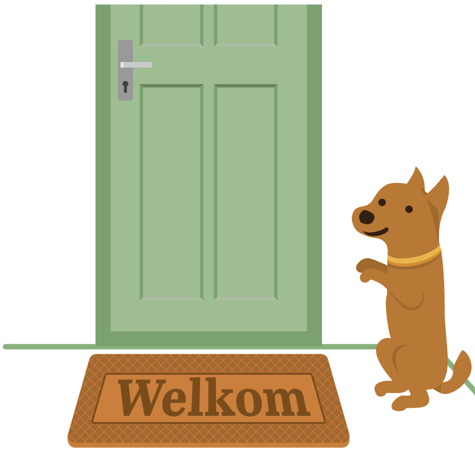

Mantelzorgers vertellen:

- Ons huisdier is graag in de buurt van mij en mijn partner.
- Zodra ik mijn partner troost, wringt ons huisdier zich ertussen.
- Ons huisdier richt zich meer op mijn partner dan op mij.
- Ons huisdier weet wanneer mijn partner thuiskomt... en wacht hem op.
- Ik voer hele gesprekken met mijn huisdier.

Zorgverleners vertellen:

- Ik merk dat huisdieren ook mijn aandacht vragen wanneer ik bij een cliënt kom.
- Ik zie dat het huisdier anders reageert als de cliënt pijn heeft.
- Door hoe een cliënt omgaat met het huisdier, merk ik dat er iets aan de hand is.
- Ik zie dat het huisdier de cliënt in de gaten houdt.
- Ik merk dat knuffelen met een huisdier veel doet voor mensen met dementie.

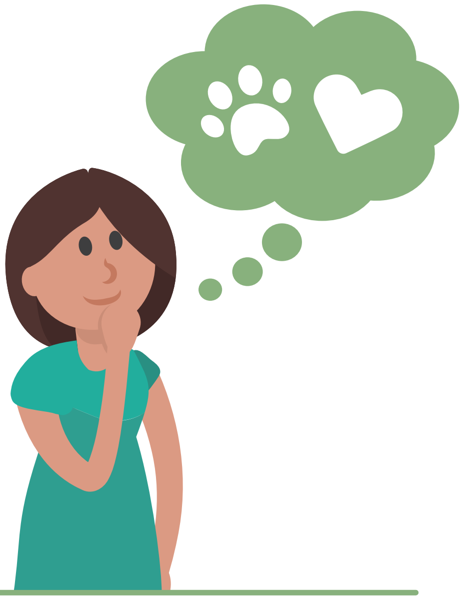

Dit project is mede mogelijk gemaakt door:

ZonMw
Open Universiteit
Radboudumc
UKON Universitair Kennisnetwerk Ouderenzorg Nijmegen
de Zorgboog voor alle generaties

De band met het huisdier

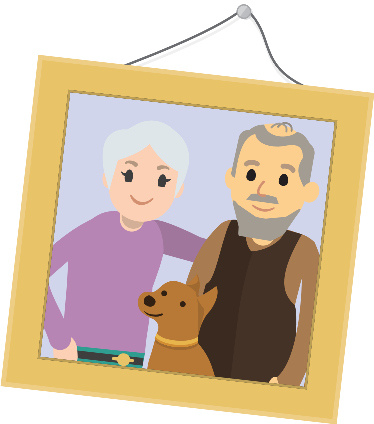

Hechting

- Cliënten ervaren vaak een sterke band met hun huisdier.
- Cliënten beschouwen hun huisdier als een vriend en als onderdeel van het gezin.
- Cliënten betrekken hun huisdier bij activiteiten.
- Cliënten vinden het moeilijk wanneer ze afstand moeten nemen van hun huisdier.

Onvoorwaardelijke liefde

- Cliënten ervaren de liefde van hun huisdier als onvoorwaardelijk.
- Een huisdier stelt geen lastige vragen, oordeelt niet, vertelt niets door en is altijd beschikbaar.
- Cliënten kunnen zichzelf zijn bij hun huisdier.

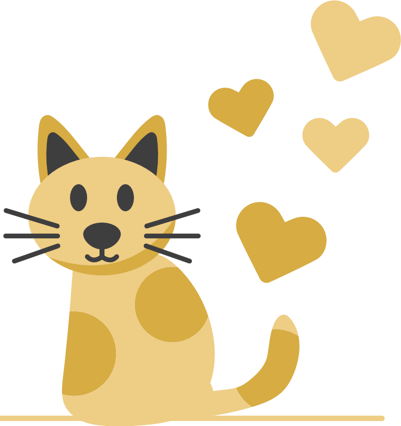

Wederzijdse afhankelijkheid

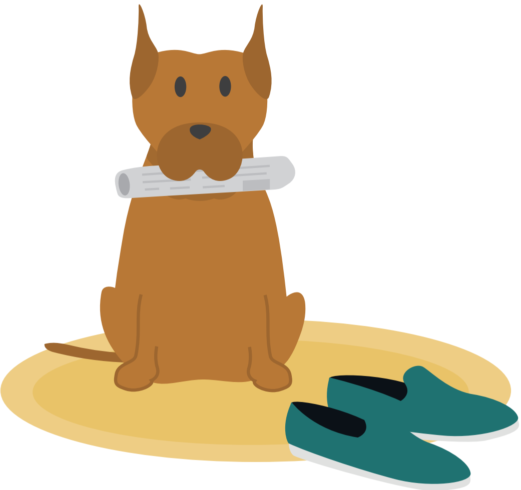

- Cliënten zorgen voor hun huisdier, maar huisdieren ondersteunen ook hun baasje.
- Een huisdier kan een cliënt door een moeilijke situatie heen helpen.
- Een huisdier kan een leegte opvullen en zorgt ervoor dat een cliënt zich goed voelt.

Een mantelzorger vertelt:

'Ons huisdier is als een kind voor ons.'

PET@home
Als het baasje zorg nodig heeft

Cliënten vertellen:

- Mijn huisdier is mijn vriend.
- Ik merk dat mijn huisdier ook van mij houdt.
- Ik voel dat ik geaccepteerd word door mijn huisdier.
- Ik bespaar vaak op andere kosten, zodat ik geld over heb voor een dierenartsbehandeling.

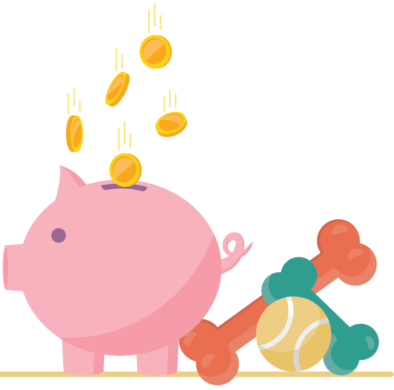

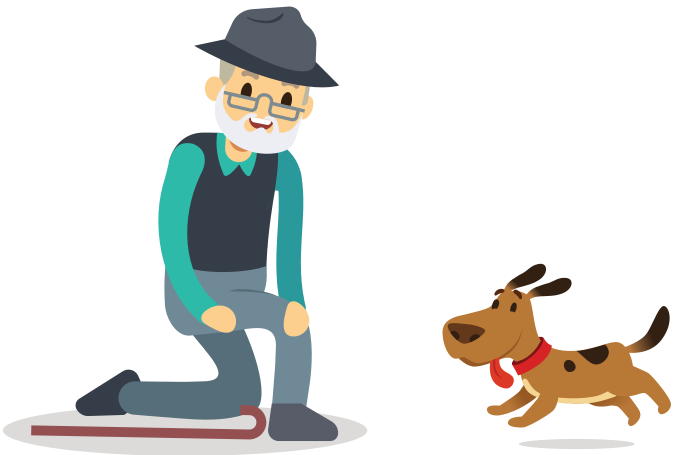

Mantelzorgers vertellen:

- Mijn partner heeft een sterke band met ons huisdier.
- Ik heb zelf ook een sterke band met het huisdier.
- De band met ons huisdier betekent veel voor mijn partner.

Zorgverleners vertellen:

- Ik zie dat de band tussen cliënt en huisdier vaak heel sterk is.
- Ik merk dat de band tussen cliënt en huisdier sterker is wanneer de cliënt de directe verzorger is.
- Het huisdier vertrouwt de cliënt.
- Het huisdier biedt veiligheid.

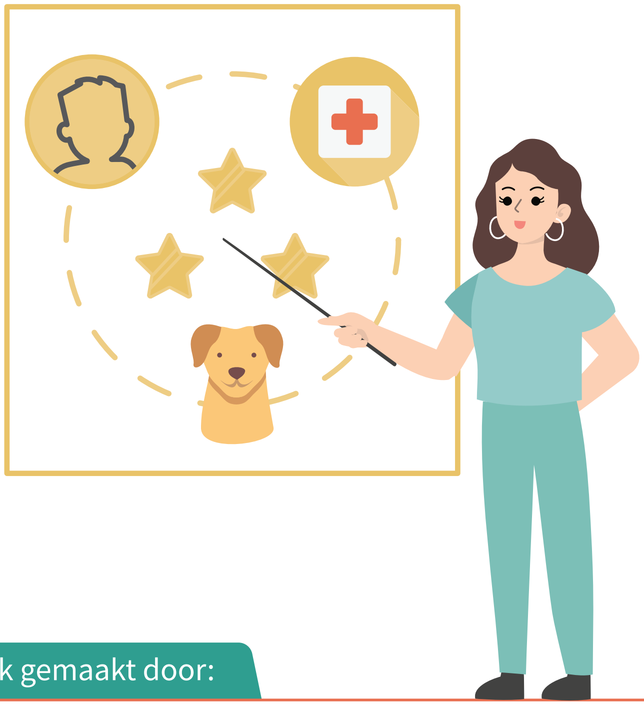

Dit project is mede mogelijk gemaakt door:

ZonMw | Open Universiteit | Radboudumc | UKON Universitair Kennisnetwerk Ouderenzorg Nijmegen | de Zorgboog voor alle generaties

Overtuigingen en herinneringen

Gevoelens herkennen

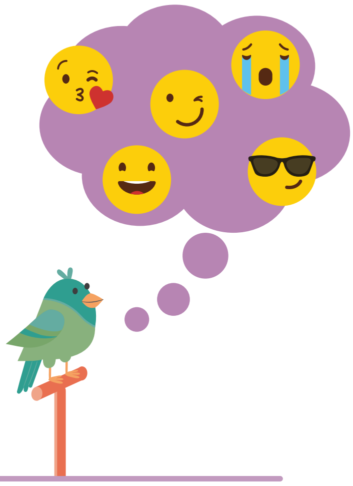

- Elk huisdier heeft een eigen persoonlijkheid.
- Cliënten geven aan verschillende emoties in hun huisdier te herkennen.
- Cliënten ervaren dat hun huisdier hen begrijpt.
- Sommige huisdieren begroeten hun baasje wanneer deze even is weggeweest.

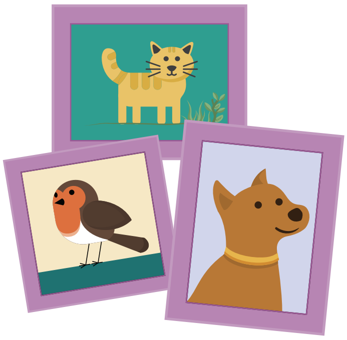

Herinneringen

- Cliënten hebben herinneringen aan hun huisdieren.
- Cliënten denken terug en praten over huisdieren die ze vroeger gehad hebben.
- Cliënten zetten foto's in huis neer als aandenken aan hun overleden huisdier.

Voldoening

- Cliënten vinden het zorgen voor hun huisdier een fijne en nuttige invulling van de dag.
- Cliënten krijgen door hun huisdier het gevoel dat ze ergens bij horen.
- Een huisdier kan een positieve invloed hebben op het zelfvertrouwen en de eigenwaarde van een cliënt.

Betekenis van het leven

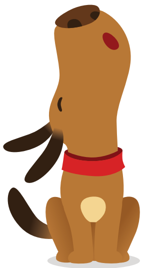

- Cliënten vinden dat ze er voor hun huisdier moeten en bovendien willen zijn.
- Een huisdier geeft invulling aan het leven van een cliënt.
- Sommige cliënten zeggen dat ze niet zonder een huisdier willen en misschien ook wel niet kunnen leven.

Een zorgverlener vertelt:

'Als deze cliënt dat hondje niet had, dan stond ze 's morgens niet op, dan hoefde het voor haar niet.'

PET@home
Als het baasje zorg nodig heeft

Cliënten vertellen:

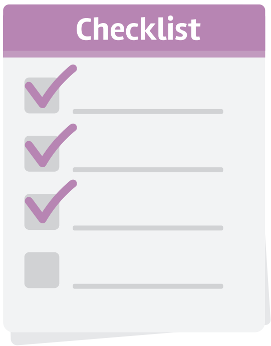

- Ik besteed nu meer aandacht aan mijn huisdier dan aan eerdere huisdieren. Ik heb nu meer tijd dan vroeger.
- Ik vind het fijn als een ander mijn huisdier aandacht geeft.
- Ik voel dat ik er nog toe doe, doordat ik van alles moet regelen voor mijn huisdier.
- Ik heb herinneringen aan mijn (eerdere) huisdieren en praat hier graag over.
- Ik herdenk mijn eerdere huisdieren rondom de sterfdatum.

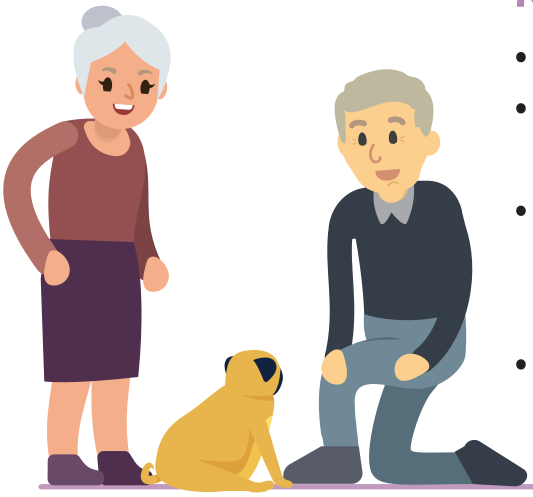

Mantelzorgers vertellen:

- Een huisdier kan mijn leven dragelijker maken.
- Een huisdier geeft invulling aan mijn leven en is een prettige tijdsbesteding.
- Ik merk dat mijn partner zich betrokken voelt bij het huishouden als hij kan helpen zorgen voor ons huisdier.
- Ik zie dat mijn partner zichzelf verwaarloost wanneer hij geen huisdier meer heeft.

Zorgverleners vertellen:

- Ik merk dat een huisdier vaak alles is voor de cliënt.
- Cliënten voelen zich door het huisdier weer nuttig en dit heeft een positieve invloed op hun eigenwaarde.
- Sommige cliënten zeggen dat het leven niet meer hoeft zonder huisdier.

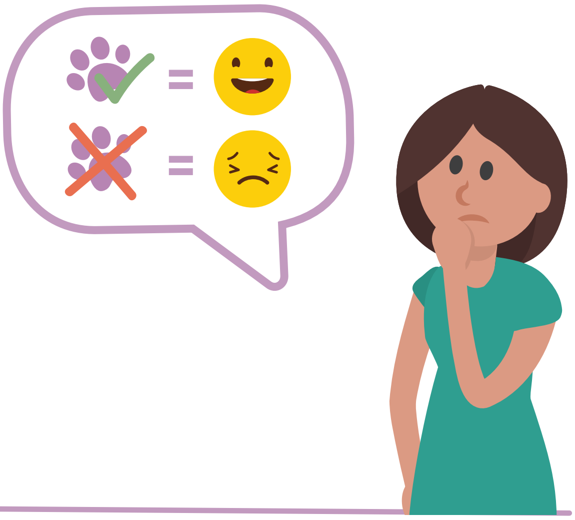

Dit project is mede mogelijk gemaakt door:

ZonMw · Open Universiteit · Radboudumc · UKON Universitair Kennisnetwerk Ouderenzorg Nijmegen · de Zorgboog voor alle generaties

Emotionele gezondheid

Reageren op gevoelens

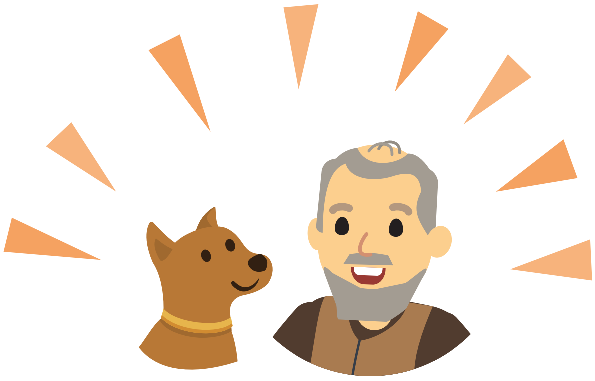

- Cliënten en huisdieren reageren op elkaars gevoelens.
- De vrolijkheid van het huisdier werkt vaak aanstekelijk. Hun baasjes worden ook vrolijker.

Emotionele steun

- Een huisdier biedt veel steun aan cliënten.
- Cliënten zeggen dat ze het gevoel hebben dat het huisdier ze troost wanneer ze zich niet goed voelen.
- Een huisdier vult een bepaalde leegte en biedt houvast.

Plezier

- Cliënten lachen vaak om hun huisdier en beleven zo veel plezier.
- Een huisdier zorgt voor gezelligheid en levendigheid in huis.
- Cliënten nemen hun huisdier vaak mee op uitjes buitenhuis, bijvoorbeeld op bezoek bij vrienden of op vakantie.

Rouw en verdriet

- De dood van een huisdier kan bij cliënten een heftig rouwproces in gang zetten.

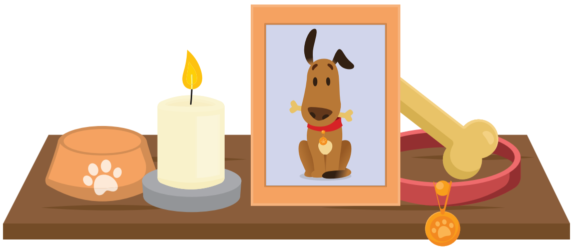

- Veel cliënten ervaren een emotioneel moment wanneer ze afscheid moeten nemen van hun huisdier.
- Sommige cliënten organiseren een begrafenis of crematie voor hun huisdier.
- Veel cliënten zetten bijvoorbeeld foto's in huis neer om hun overleden huisdier te herdenken.

Cliënten vertellen:

- Mijn huisdier merkt wanneer ik niet goed in mijn vel zit.
- Mijn huisdier zoekt mij op en hierdoor voel ik mij weer wat beter.
- Ik geniet wanneer ik zie dat mijn huisdier vrolijk is.
- Ik ervaar de dood van mijn huisdier als een verschrikkelijke gebeurtenis.
- Ik mis vooral de gezelligheid na de dood van mijn huisdier en ik wil dan ook meteen een nieuw huisdier.

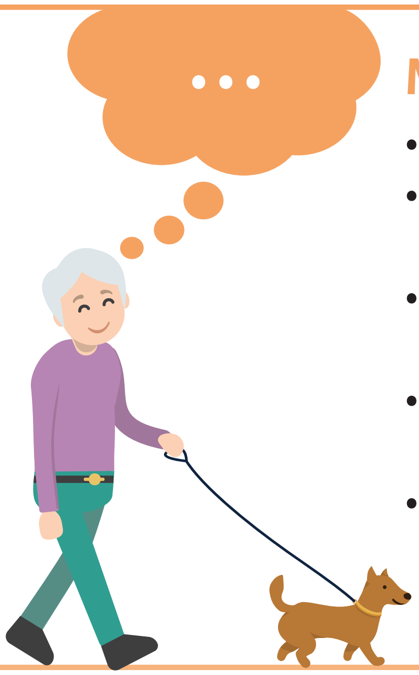

Mantelzorgers vertellen:

- Het huisdier voelt mijn stemming aan.
- Het huisdier zorgt ervoor dat ik mij snel weer wat vrolijker voel.
- Ik kan mijn hoofd leegmaken tijdens het uitlaten van de hond.
- Ik vind het leuk om samen met mijn partner onze hond uit te laten.
- Ik zie dat ons huisdier merkt dat de gezondheid van mijn partner achteruitgaat en het gedrag hierop aanpast.

Zorgverleners vertellen:

- Ik praat graag met cliënten over hun huisdier. Het is niet zo'n beladen onderwerp en kan de sfeer verbeteren.
- Ik ondersteun cliënten na het overlijden van hun huisdier. Voor cliënten valt er dan een groot deel van het leven weg, zoals bepaalde zorgtaken voor het huisdier en de structuur.
- Ik zie dat een huisdier gezelligheid biedt en dat dit belangrijk is voor cliënten.

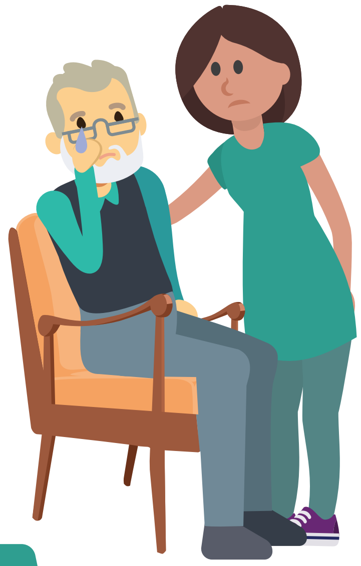

Dit project is mede mogelijk gemaakt door:

ZonMw · Open Universiteit · Radboudumc · UKON Universitair Kennisnetwerk Ouderenzorg Nijmegen · de Zorgboog voor alle generaties

De verzorging van het huisdier

Behoefte om te zorgen

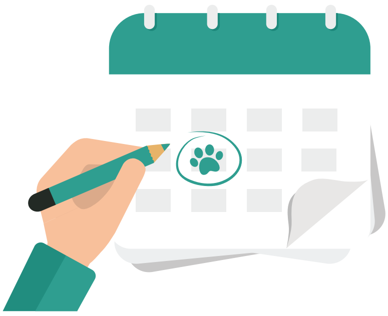

- Cliënten vinden het prettig om voor een huisdier te zorgen en om hun huisdieren te verwennen.
- Cliënten maken vaak afspraken met anderen rondom de zorg voor het huisdier.

Verantwoordelijkheid

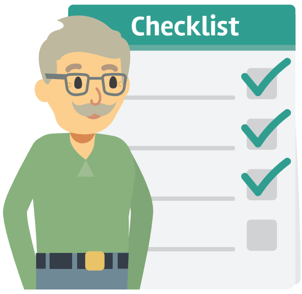

- Cliënten vinden dat ze moeten zorgen voor hun huisdier.
- Cliënten vinden het soms lastig dat ze dingen moeten regelen voor hun huisdier, bijvoorbeeld als ze een dagje weg willen.
- Cliënten vinden het belangrijk om verantwoordelijkheid te nemen wanneer ze, bijvoorbeeld vanwege ziekte, afscheid moeten nemen van hun huisdier.

Veiligheid

- Een huisdier geeft een cliënt een gevoel van veiligheid en geborgenheid.
- Sommige honden blaffen en grommen. Dit kan cliënten een veilig gevoel geven.

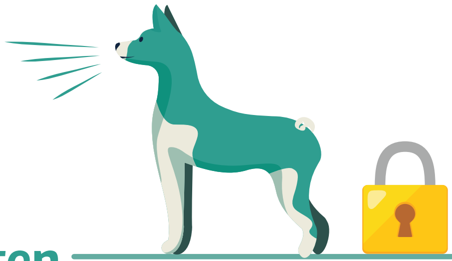

Kosten

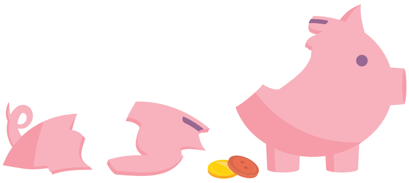

- Huisdieren kosten geld, bijvoorbeeld voor voer, de dierenarts of kapotgebeten meubels.
- Cliënten kunnen deze kosten als een belemmering ervaren om voor een huisdier te zorgen.

Zorgen

- Cliënten maken zich ongerust over hun huisdier. Dit kan gaan over hun gezondheid, het mogelijk overlijden of het weglopen van hun huisdier.
- Cliënten maken zich ook ongerust over het moeten achterlaten van het huisdier, bijvoorbeeld als ze naar een verpleeghuis gaan.

Een mantelzorger vertelt:

'De hond beschermt mijn vrouw. Dat is echt ongelofelijk.'

PET@home
Als het baasje zorg nodig heeft

Cliënten vertellen:

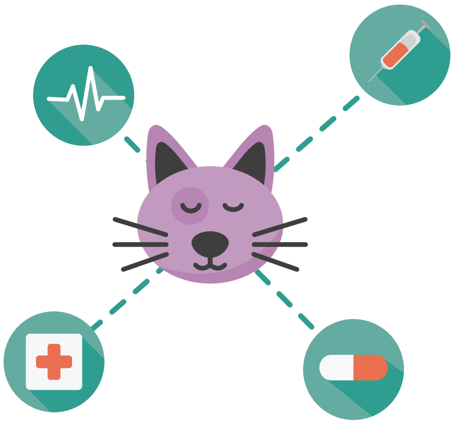

- Ik zorg graag voor mijn huisdier en merk dat mijn huisdier ook voor mij zorgt.
- Ik vind het fijn om bezig te zijn en voor mijn huisdier moet van alles geregeld worden.
- Ik stel de gezondheid van mijn huisdier voorop.
- Ik maak mij ongerust over wat er gebeurt met mijn huisdier wanneer ik er zelf niet meer voor kan zorgen.

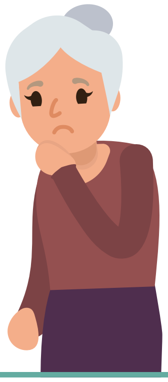

Mantelzorgers vertellen:

- Ik help vaak met de zorg voor het huisdier. Ik laat de hond uit en ga mee naar de dierenarts.
- Naarmate mijn partner verder achteruitgaat, krijg ik steeds meer verantwoordelijkheid voor de zorg van ons huisdier.
- Ik merk dat het huisdier steeds meer een belemmering begint te vormen.

Zorgverleners vertellen:

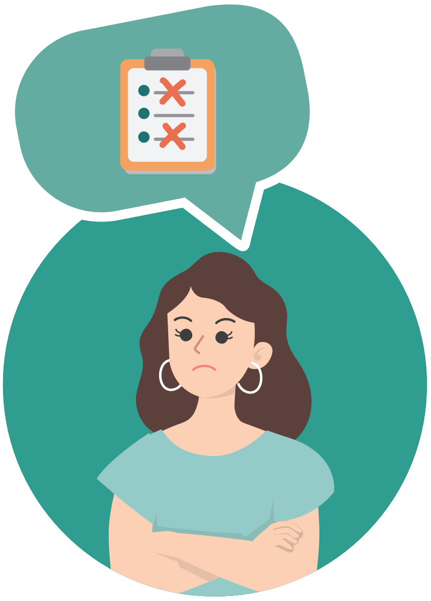

- Wanneer een cliënt niet meer goed zelfstandig voor het huisdier kan zorgen, ontstaan er problemen.
- Ik ga het gesprek aan als ik merk dat het zorgen voor het huisdier niet meer lukt.
- Ik merk dat cliënten en naasten wisselend reageren op mijn advies, maar de verantwoordelijkheid ligt bij hen.
- Huisdieren beïnvloeden soms zorgbeslissingen. Cliënten stellen bijvoorbeeld verhuizing naar een verpleeghuis uit of willen niet naar een dagopvang, omdat het huisdier dan alleen is.
- Huisdieren geven een doel aan cliënten en zijn daarom een meerwaarde.

Dit project is mede mogelijk gemaakt door:

ZonMw | Open Universiteit | Radboudumc | UKON Universitair Kennisnetwerk Ouderenzorg Nijmegen | de Zorgboog voor alle generaties
