# Supplementary material for: The development of the PET@home toolkit: An experience-based co-design method study
Source: Int J Nurs Stud Adv. 2024 Mar 6;6:100189. doi: 10.1016/j.ijnsa.2024.100189 (PMC11080344; doi:10.1016/j.ijnsa.2024.100189)
Supplement: Supplementary file 4 [file mmc4.pdf]

PET@home Toolkit

PET@home
Als het baasje zorg nodig heeft

Waarom de Toolkit?

De Toolkit is voor cliënten met huisdieren, hun familie en zorgmedewerkers en draagt bij aan:

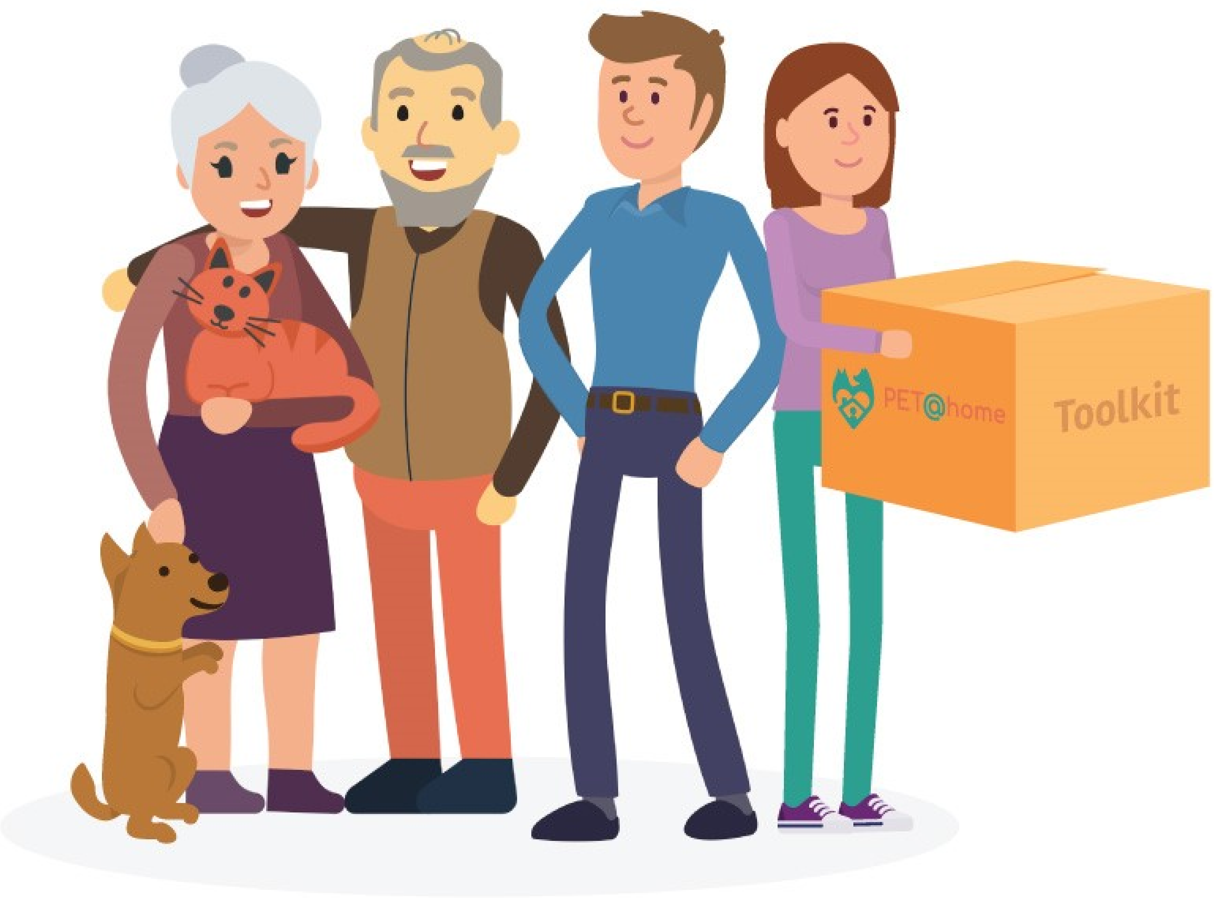

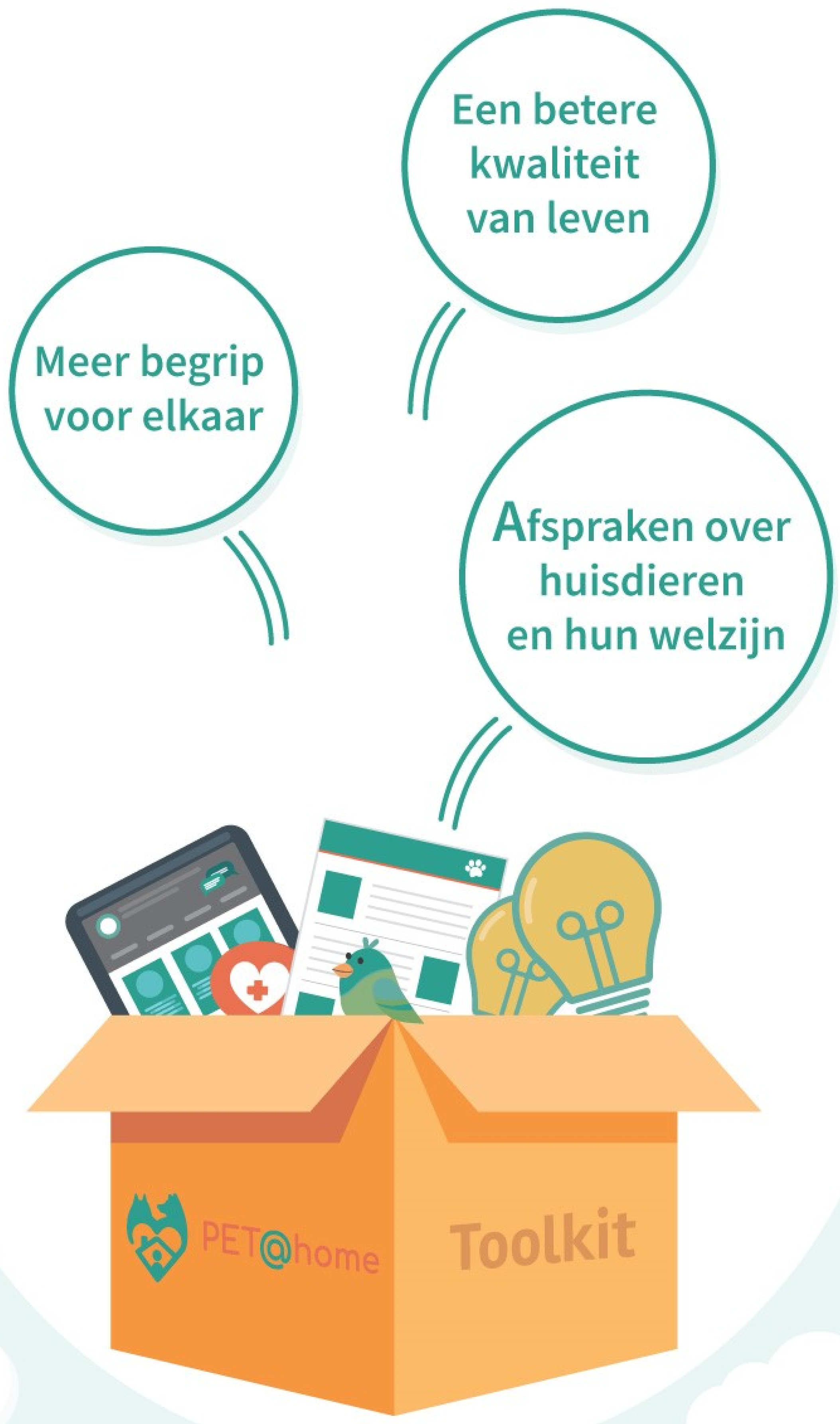

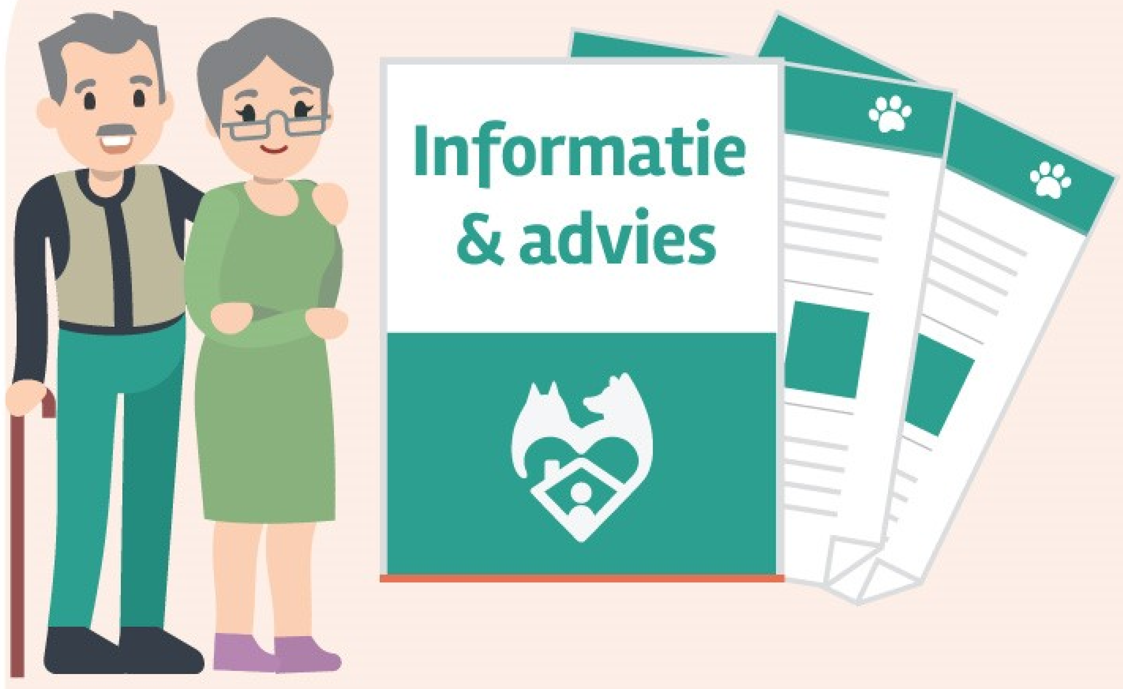

Informatiebrochure

Lees waarom huisdieren belangrijk zijn en welke uitdagingen er kunnen zijn. Gebruik de checklist om afspraken vast te leggen.

Voor wie?
Zorgmedewerkers
Cliënten en naasten

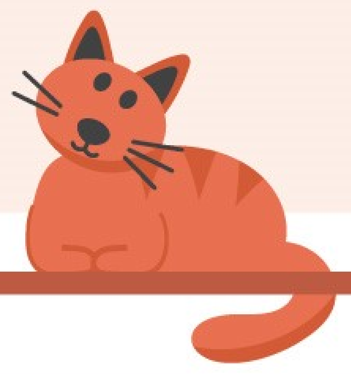

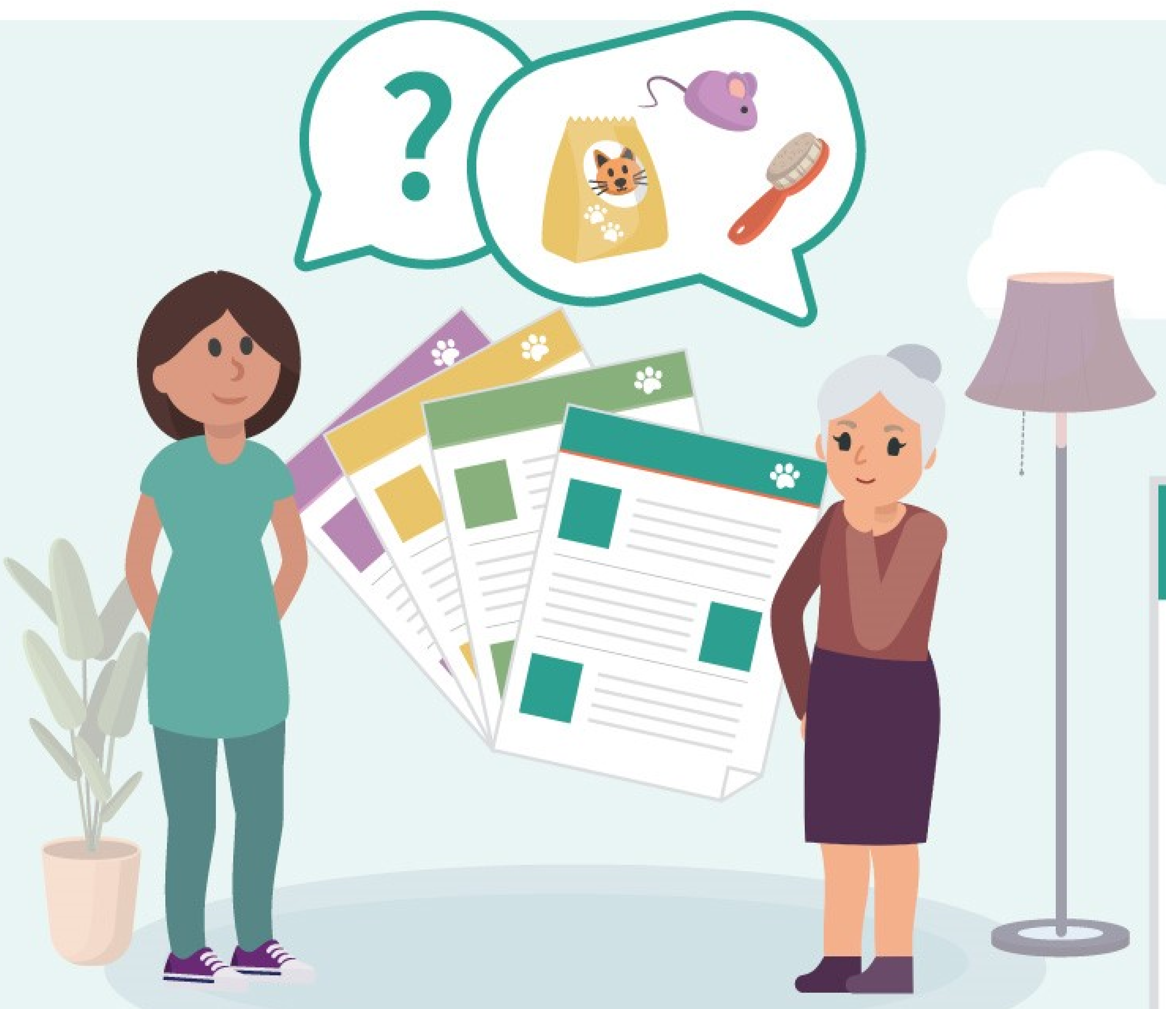

Gesprekskaarten

Gebruik de kaarten om in gesprek te gaan over huisdieren en om elkaar beter te begrijpen

Voor wie?
Zorgmedewerkers

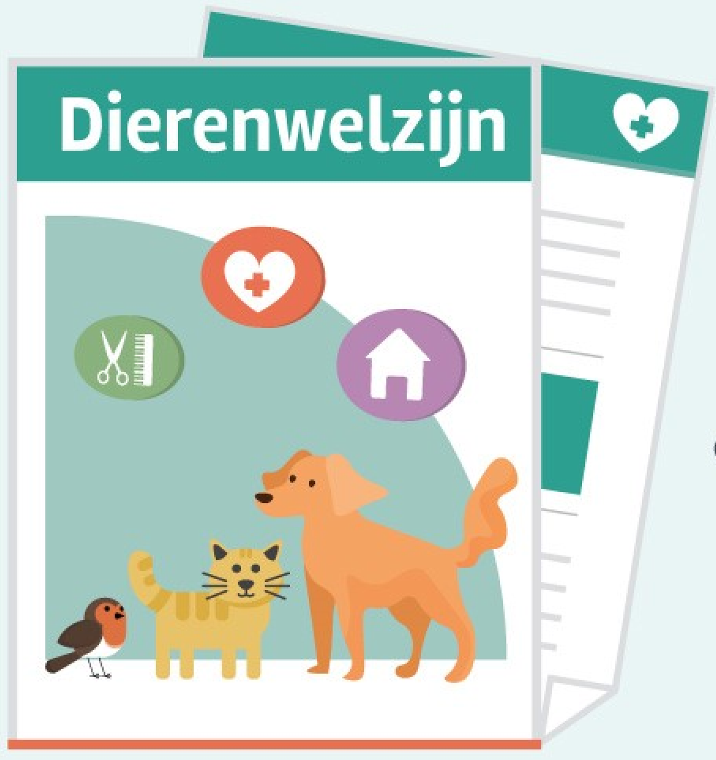

Leaflet Dierenwelzijn

Lees over het welzijn van het huisdier en wat te doen als er iets niet goed gaat

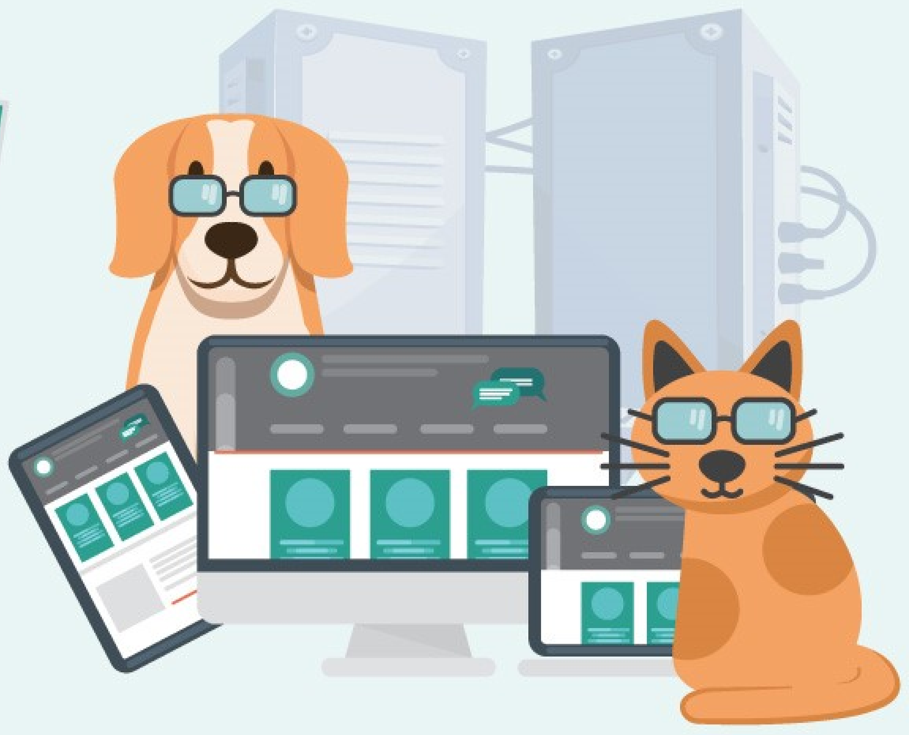

E-learning

Meld je aan voor cursus CODE www.ou.nl en leer over de rol van huisdieren voor cliënten.

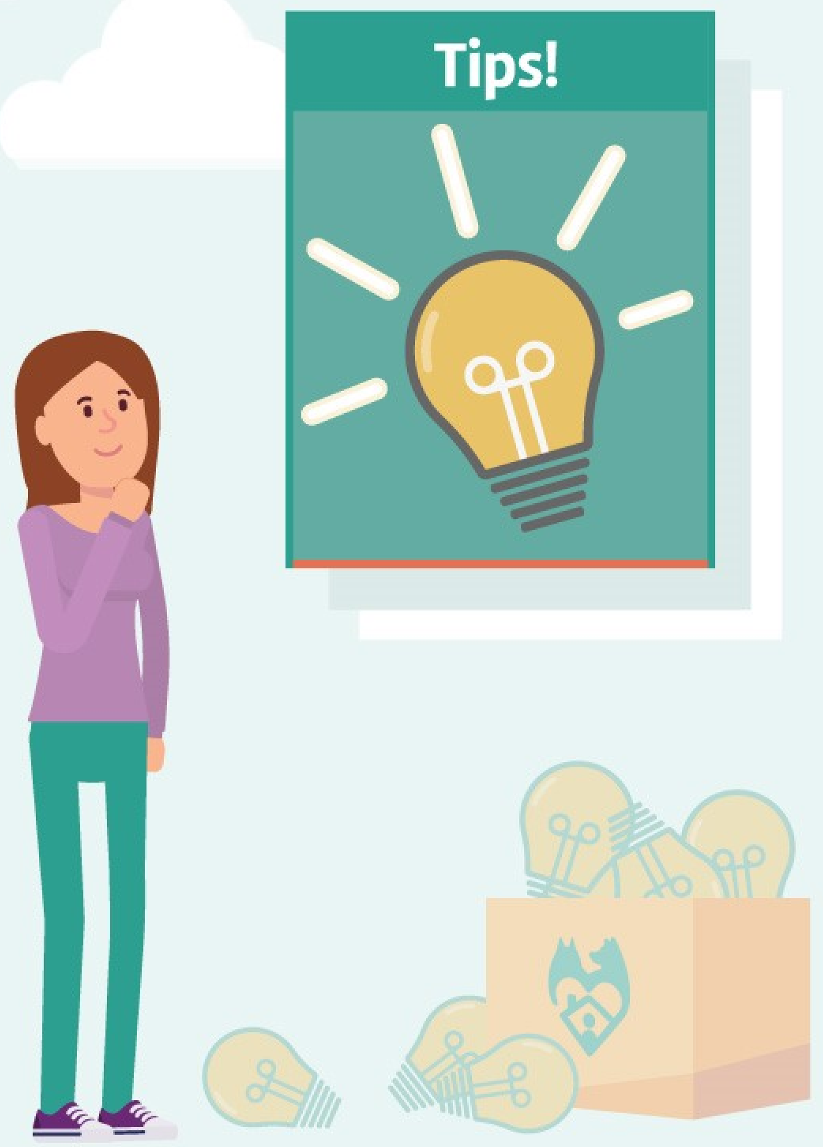

Communicatietips zorgmedewerkers

Gebruik de tips in gesprekken met cliënten

Implementatiewijzer

Gebruik dit om de Toolkit in de organisatie uit te rollen.

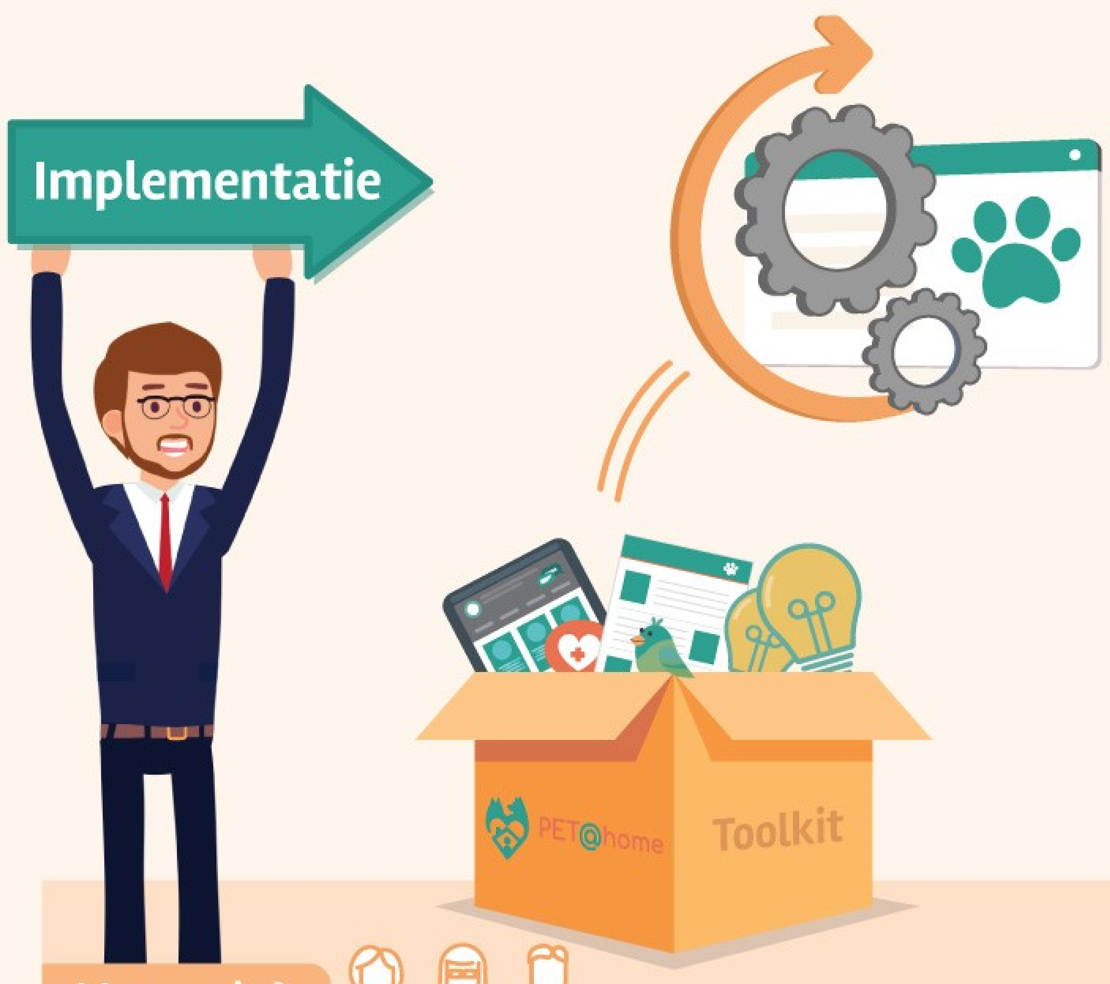

Voor wie?
Zorgmanager
Projectcoördinator

Inventarisatielijst zorgplangesprekken

Inventariseer aandachtsgebieden over het huisdier tijdens zorgplangesprekken

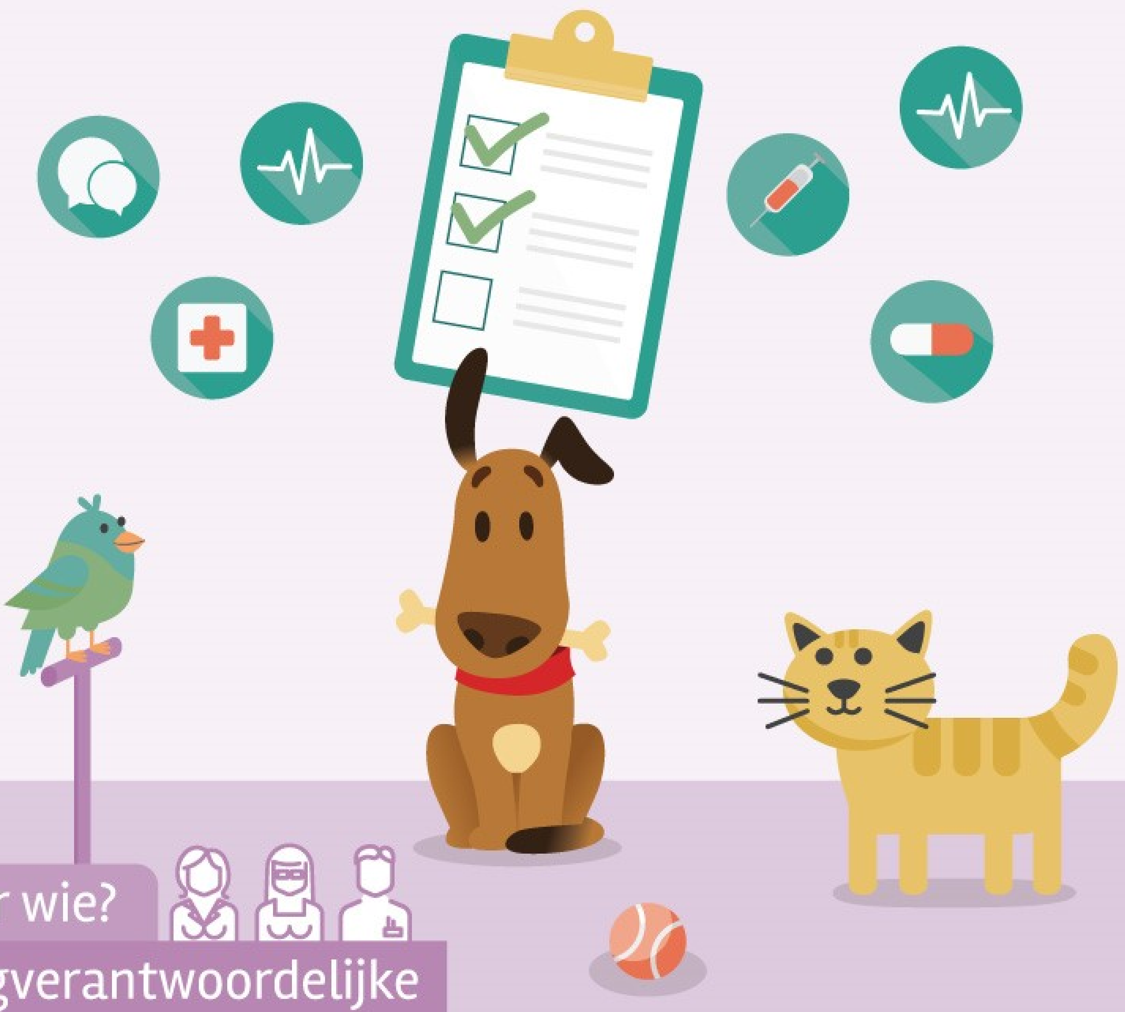

Voor wie?
Zorgverantwoordelijke
Zorgmedewerkers

Praatplaat

Hang dit op een zichtbare plek zodat iedereen weet van de Toolkit

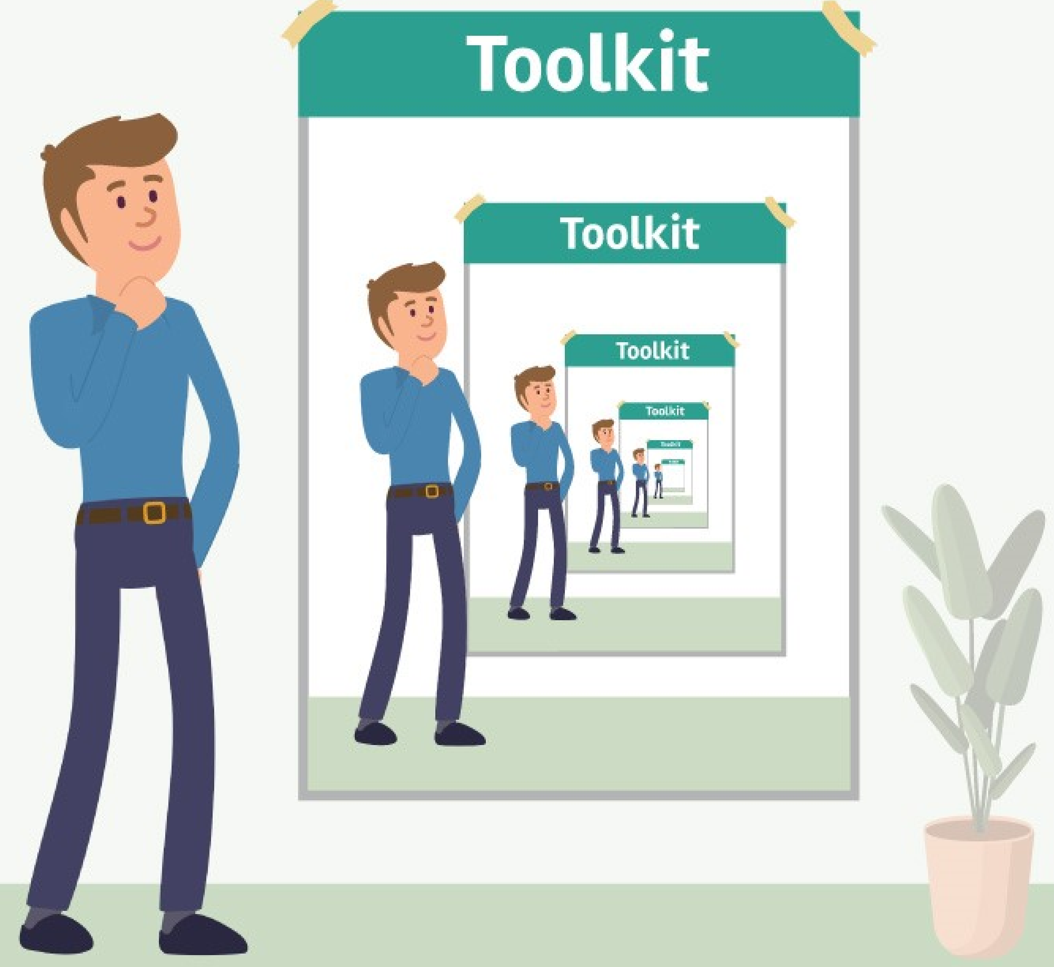

Voor wie?
Alle belanghebbenden

ZonMw
Open Universiteit
Radboudumc
UKON Universitair Kennisnetwerk Ouderenzorg Nijmegen
de Zorgboog voor alle generaties
PET@home

© 2023
