# Supplementary material for: The development of the PET@home toolkit: An experience-based co-design method study
Source: Int J Nurs Stud Adv. 2024 Mar 6;6:100189. doi: 10.1016/j.ijnsa.2024.100189 (PMC11080344; doi:10.1016/j.ijnsa.2024.100189)
Supplement: Supplementary file 5 [file mmc5.pdf]

Informatie en advies

voor personen die huisdieren hebben en thuis zorg ontvangen

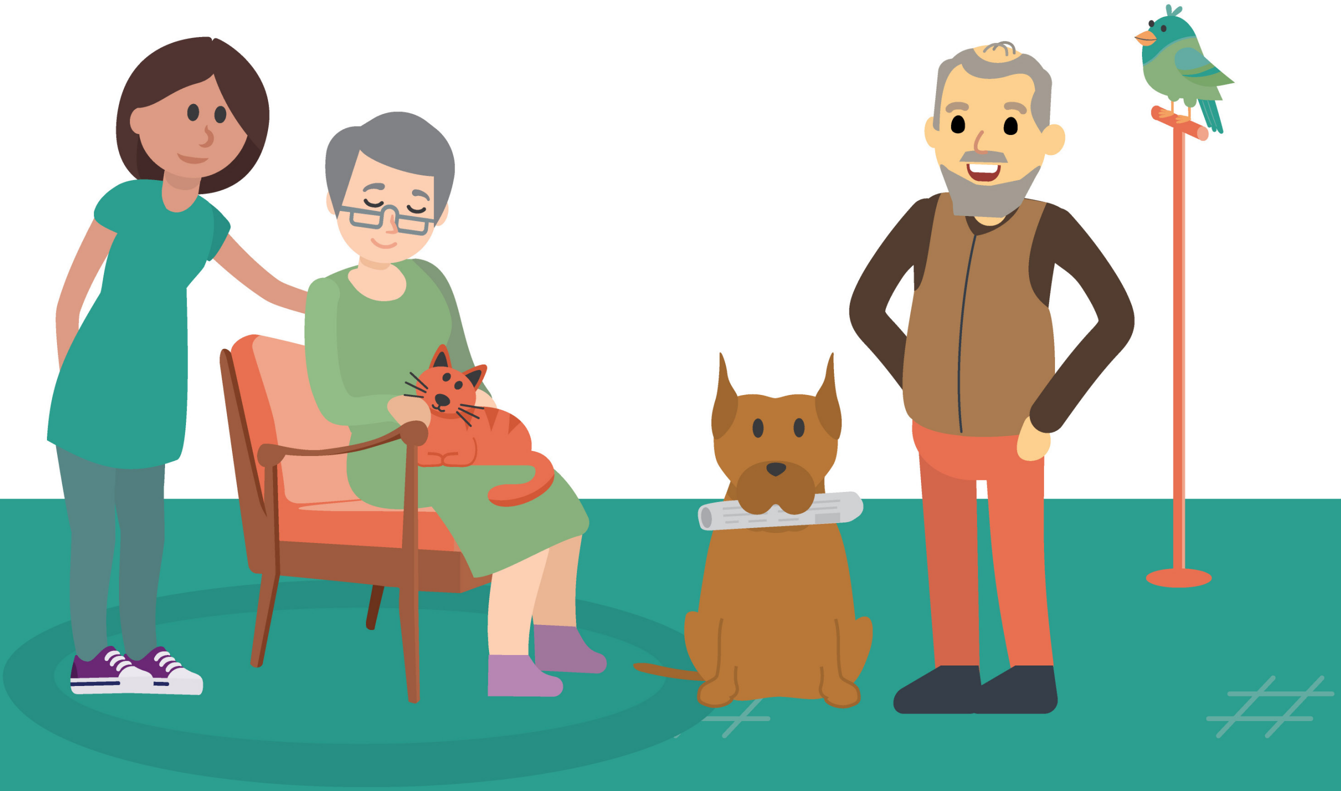

PET@home

Als het baasje zorg nodig heeft

Colofon

Deze brochure is een uitgave van de Open Universiteit.
De Open Universiteit is niet aansprakelijk voor drukfouten of omissies.

Redactie	Peter Reniers, Ine Declercq,
	Dr. R. Leontjevas, Manon Vogel
Eindredactie	Manon Vogel Tekst & Communiicatie
Hoofdredactie	Peter Reniers
Vormgeving & Illustratie	Tomas Herder

Deze brochure is tot stand gekomen met medewerking van zorgontvangers, mantelzorgers en zorgmedewerkers.

© 2023

Informatie en advies voor personen die huisdieren hebben en thuis zorg ontvangen

Ruim de helft van alle huishoudens in Nederland heeft gezelschapsdieren. Huisdieren kunnen een positieve invloed hebben op het leven en welzijn van mensen.

Als u thuis zorg ontvangt, komt uw dier in contact met de zorgmedewerker(s). Dit kan effect hebben op zowel de zorgrelatie als uw huisdier.

Deze brochure bevat informatie over huisdieren bij personen die thuiszorg ontvangen en bevat praktische tips en adviezen voor hun baasjes.

Inhoudsopgave

1. **Uw huisdier en thuiszorg**
   Verantwoordelijkheden en taakverdeling
2. **Uw zorgmedewerker**
   Tips voor communicatie over uw huisdier
3. **Daarom is uw huisdier belangrijk!**
   Handvatten voor een gesprek over uw huisdier
4. **Het welzijn van uw huisdier**
   Omgevingsverrijking voor dieren van zorgontvangers
5. **Ondersteuningsmogelijkheden**
   Extra handen bij de zorg voor uw huisdier
6. **PET@Home checklist**
   Afspraken voor huisdierbezitters en hun naasten
7. **Afscheid**
   Over herplaatsing of overlijden van uw huisdier
8. **Een nieuw huisdier**
   Adviezen over een eventueel nieuw huisdier
9. **Handige websites en tips**
   Een overzicht van nuttige websites en zoektermen
10. **Bijlage: Afsprakenposter**
    Helderheid voor u en uw zorgmedewerker

1. Uw huisdier en thuiszorg

U heeft een (of meerdere) huisdier(en) en u ontvangt (binnenkort) zorg aan huis. Uw dier komt mogelijk ook in contact met de zorgmedewerkers die bij u thuis komen. Het is voor iedereen belangrijk dat dit contact op een harmonieuze manier verloopt.

Hieronder vindt u enkele aandachtsgebieden en adviezen rondom het contact tussen uw huisdier en zorgmedewerkers. Verderop in deze brochure vindt u een PET@Home checklist, waarmee afspraken kunnen worden vastgelegd.

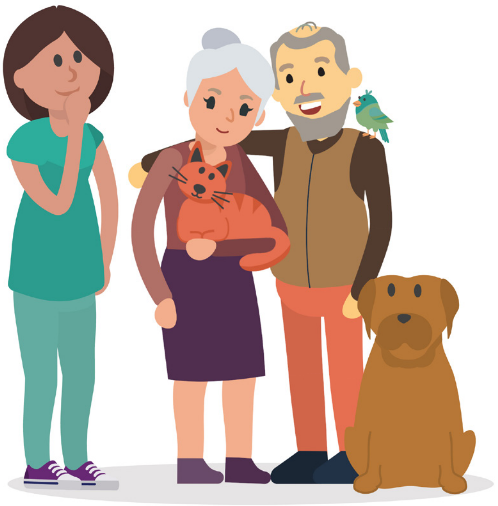

Verantwoordelijkheid

De verantwoordelijkheid om goede zorg aan uw huisdier te geven ligt bij u en uw eventuele naasten. Medewerkers van thuiszorgorganisaties voeren geen zorgtaken uit voor huisdieren van cliënten. Wel kunnen ze meedenken over oplossingen rondom uw huisdier.

Mensen die thuiszorg ontvangen, kunnen soms steeds minder goed voor hun huisdier zorgen. Voor het welzijn van zowel degene die zorg ontvangt als het huisdier zelf, is het van belang dat tijdig over deze situatie wordt nagedacht en afspraken over de verzorging worden gemaakt.

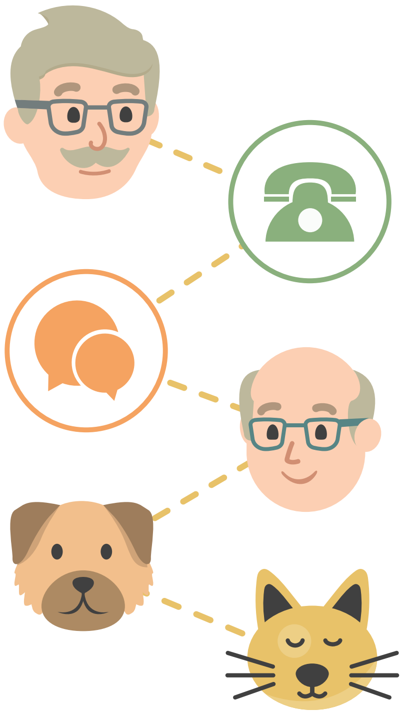

Als zorgontvangers verminderd wilsbekwaam zijn, is het wenselijk om ook een tweede contactpersoon te hebben voor het huisdier. Dit kan iemand anders zijn dan uw mantelzorger of degene die uw huisdier verzorgt.

Bespreek dit onderwerp en maak op tijd afspraken met de mensen om u heen. De namen en telefoonnummers van mede-verantwoordelijken voor de zorg van het huisdier, en die van een eventuele contactpersoon, kunnen worden genoteerd op het afsprakenformulier in deze brochure.

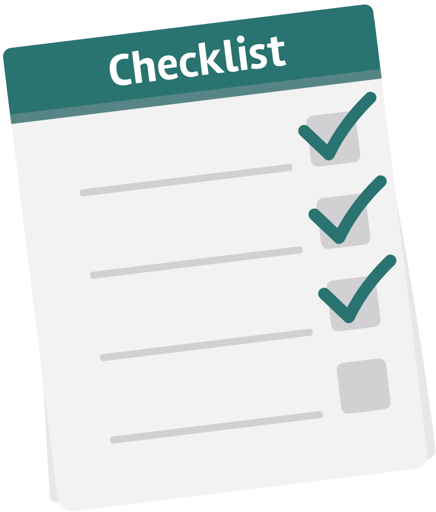

Afspraken taakverdeling

Afspraken rondom de zorg voor het huisdier bieden duidelijkheid en rust en dus minder stress. Bijvoorbeeld: U voorziet het huisdier van eten en drinken en uw mantelzorger laat het huisdier uit of maakt de bak/kooi schoon. Er kunnen ook afspraken over uw huisdier worden gemaakt met bijvoorbeeld buren of vrijwilligers, dit kan uw mantelzorger ontlasten. De afspraken kunnen ook van belang zijn voor uw zorgverlener. Leg daarom de taakverdeling rondom uw huisdier vast (zie checklist verderop in de brochure).

Bespreek regelmatig of de taakverdeling nog realistisch is. Als uw situatie verandert, verandert mogelijk ook de taakverdeling rondom uw huisdier.

Er bestaan ook praktische en technische oplossingen die u kunnen ondersteunen. Denk hierbij aan stickers of posters, zorgrobots, een dementieklok of ‘slimme’ spreekprogramma’s als extra herinnering aan zorgtaken voor het huisdier. Kijk voor meer informatie hierover in hoofdstuk 5.

Het tijdelijk, langdurig of permanent overnemen van zorg voor het huisdier

Het kan zijn dat iemand anders tijdelijk of langdurig de zorg voor uw huisdier moet overnemen, bijvoorbeeld in geval van ziekte, opname in een verpleeghuis of overlijden. Het loont om hier tijdig afspraken over te maken, ook al lijkt dat nog niet nodig. Voor mantelzorgers en zorgmedewerkers is het belangrijk dat duidelijk is wie de zorg voor het huisdier in zo’n situatie overneemt.

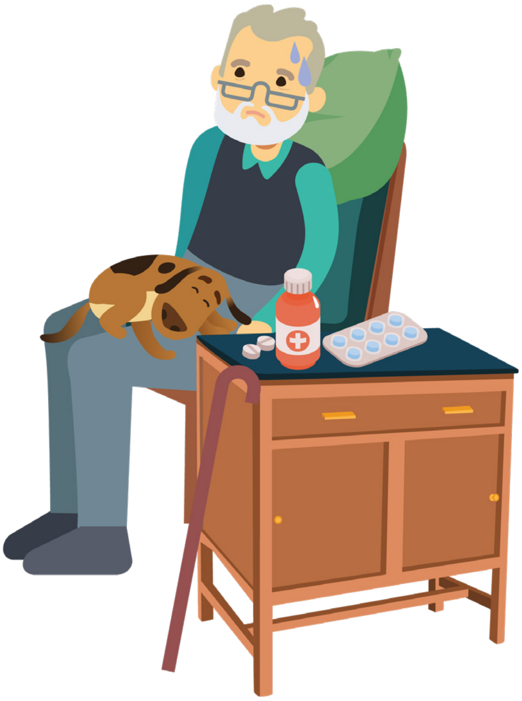

Voor het huisdier is het fijn als een tijdelijk baasje, of een nieuwe eigenaar, de gewoontes van het huisdier kent. Dit kan bij een (tijdelijke) verhuizing stress bij het huisdier verminderen. Mocht het niet mogelijk zijn om hierover afspraken te maken binnen het eigen netwerk, dan zijn er verschillende organisaties die u hierbij kunnen helpen.

Afspraken over de zorg voor uw huisdier in geval van overlijden kunnen vastgelegd worden in de checklist in deze brochure, maar ook in het levenstestament van de eigenaar.

Een huisdier bij opname in een zorginstelling

In veel gevallen biedt een zorginstelling de mogelijkheid om een huisdier mee te nemen. Hier zijn wel vaak voorwaarden aan verbonden. Het huisdier mag geen overlast veroorzaken en het baasje moet fysiek en mentaal in staat zijn het dier te verzorgen. Vaak moet iemand garant staan om de zorg voor uw huisdier over te nemen in het geval dat u dat zelf niet meer kunt. Sommige zorginstellingen laten deze zaken in een huisdierenovereenkomst vastleggen.

Mocht u uw huisdier willen meenemen naar een verpleeghuis, bespreek dan tijdig de mogelijkheden met zorginstellingen in uw omgeving.

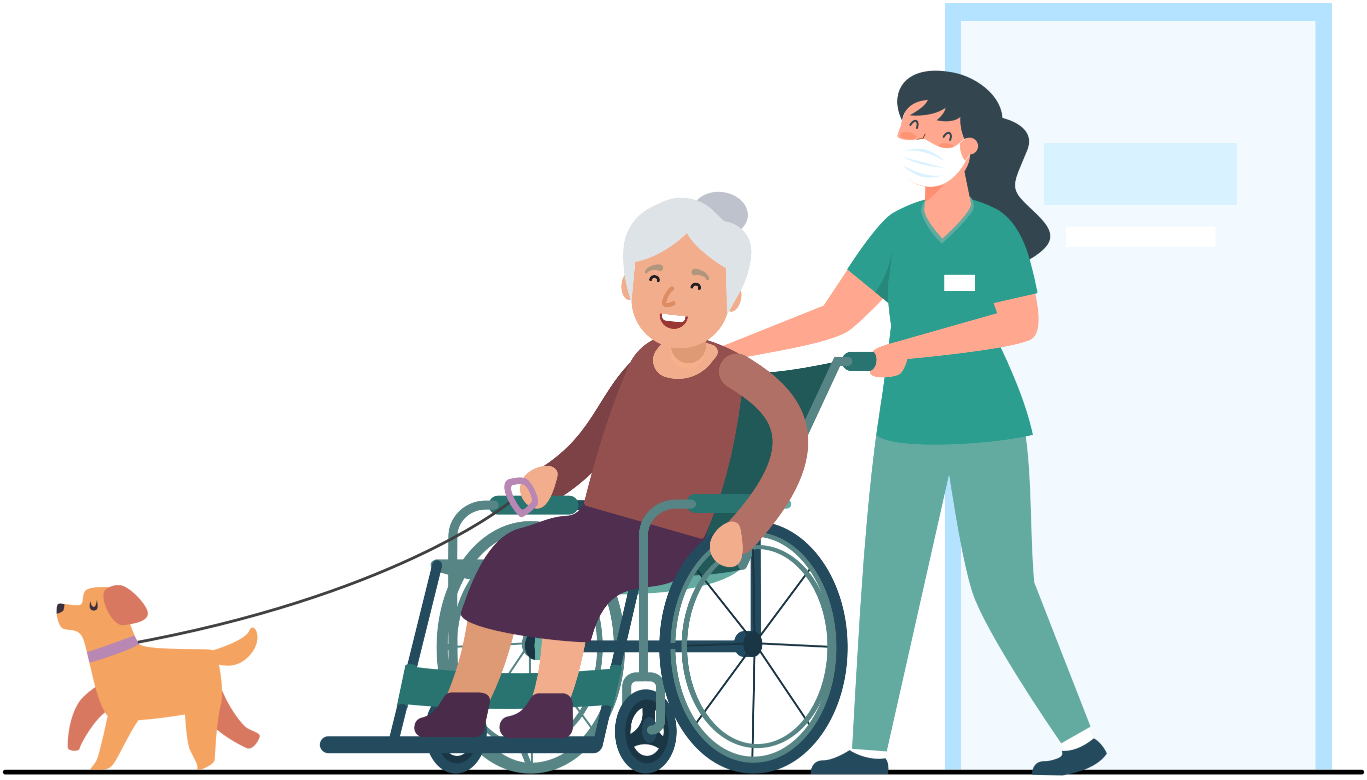

2. Uw zorgmedewerker

Vaak is het geen probleem wanneer het baasje van een huisdier thuiszorg ontvangt. Zorgmedewerkers denken graag mee, maar u blijft zelf verantwoordelijk voor uw huisdier.

Soms kan het voorkomen dat een thuiszorgmedewerker problemen heeft met de aanwezigheid van een huisdier, bv. door allergieën of gevoelens van angst. U kunt hierover afspraken maken. Zo kan uw huisdier tijdens de zorgmomenten in een andere ruimte verblijven, of kan er bij een allergie gedacht worden aan het gebruik van mondkapjes of handschoenen. Bespreek deze omgangsvormen met elkaar. Dit zal de zorgrelatie vergemakkelijken.

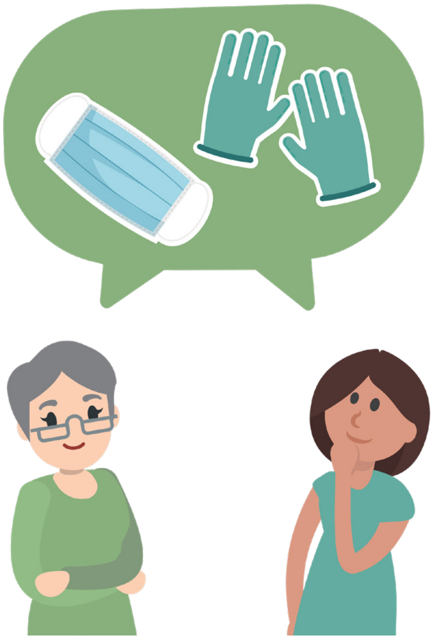

Tips om een gesprek te voeren over uw huisdier

Onderstaande tips kunnen u helpen in het gesprek met zorgverleners over uw huisdier.

1. Open vragen stellen

Uw huisdier is belangrijk voor u. Voor anderen kan uw huisdier een andere betekenis hebben. Met open vragen kunt u hier een prettig gesprek over voeren. Open vragen beginnen met vraagwoorden als wie, welke, waar, hoe en wat.

Voorbeelden: Wat vindt u van huisdieren? Waar zou u tegenaan kunnen lopen bij mijn huisdier? Hoe zouden we dat kunnen oplossen?

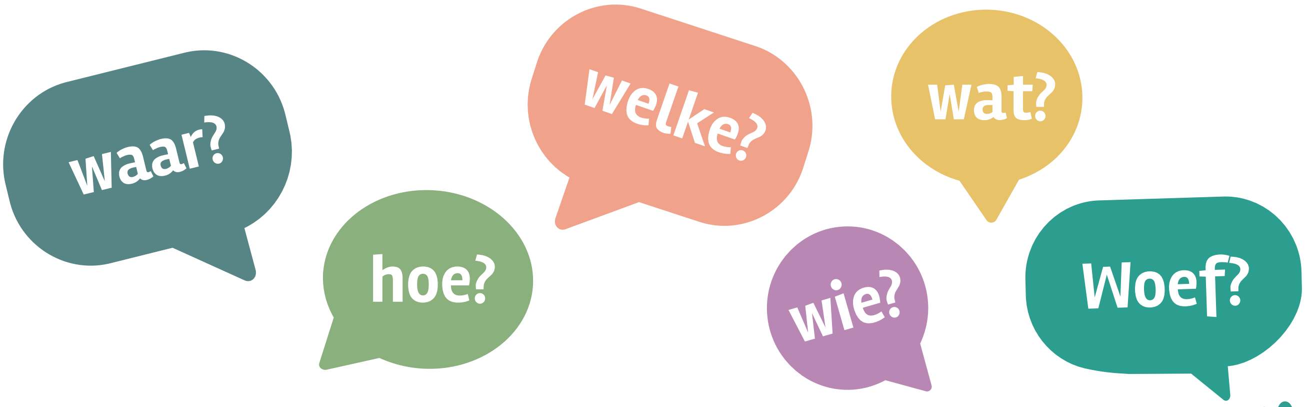

2. Begrip tonen

Iemand kan bang of allergisch zijn voor uw huisdier zonder dat u dat weet. Misschien ervaart de mantelzorger de extra zorg voor uw huisdier als belastend. Sta open voor elkaars ervaringen.

Tip: Luister naar elkaar, laat elkaar uitpraten. Heb begrip voor elkaar. Vat samen wat de ander heeft gezegd. Daarmee toetst u of u de ander goed begrepen hebt. Stel vragen wanneer iets niet duidelijk is.

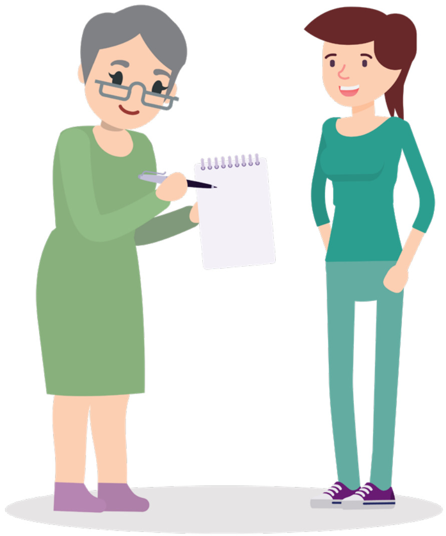

3. Afspraken delen

Leg afspraken over de zorg voor uw huisdier vast.
Zorg dat u een plan heeft waar u op kunt terugvallen in geval van nood. Deel dit plan met de zorgmedewerker(s), zodat zij hiervan op de hoogte zijn. Zo geeft u duidelijkheid.

Tip: Denk bij dit plan na over de nabije en verre toekomst. Hoe kunnen problemen worden voorkomen/opgelost? Leg afspraken vast met behulp van de checklist in deze brochure. Hang de checklist op een zichtbare plek.

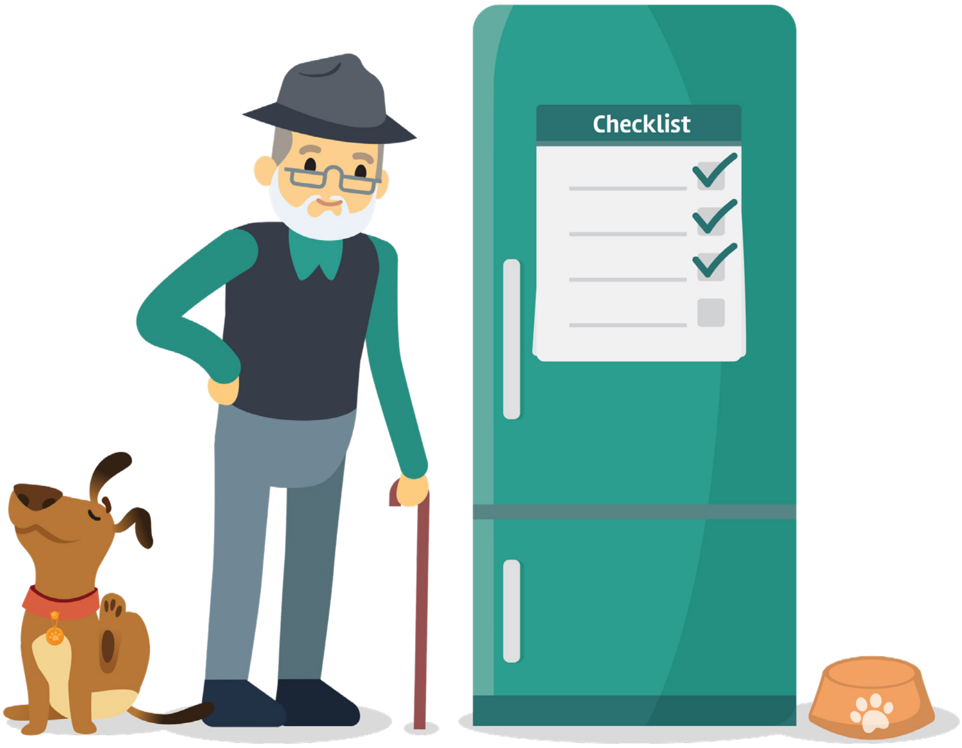

3. Daarom is een huisdier belangrijk!

Een huisdier heeft een positief effect op het welzijn van zijn of haar baasje. Gezelschapsdieren kunnen bijvoorbeeld helpen bij het tegengaan van eenzaamheid en om ouderen langer actief te laten blijven. Onderstaande feiten kunnen u helpen bij het aangaan van gesprekken over uw huisdier met anderen, bijvoorbeeld medewerkers van zorginstanties.

Fysieke gezondheid

Het hebben van een huisdier is goed voor de gezondheid. Het motiveert om meer te bewegen, zowel binnen als buiten. Huisdieren zorgen ook voor afleiding van bijvoorbeeld pijn en negatieve emoties en voor structuur en ritme. Bovendien zorgt aanraking en verzorging van een huisdier ervoor dat het stofje oxytocine vrijkomt in het lichaam. Oxytocine werkt stressverlagend en ontspannend.

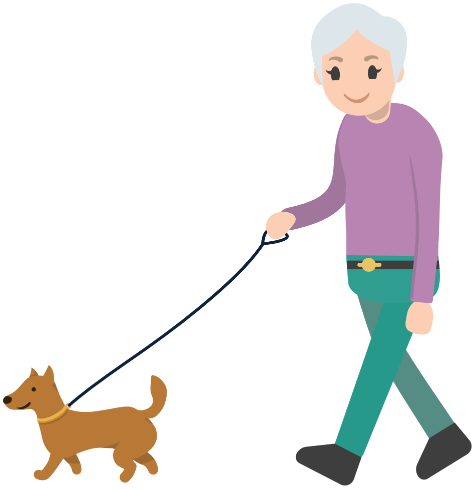

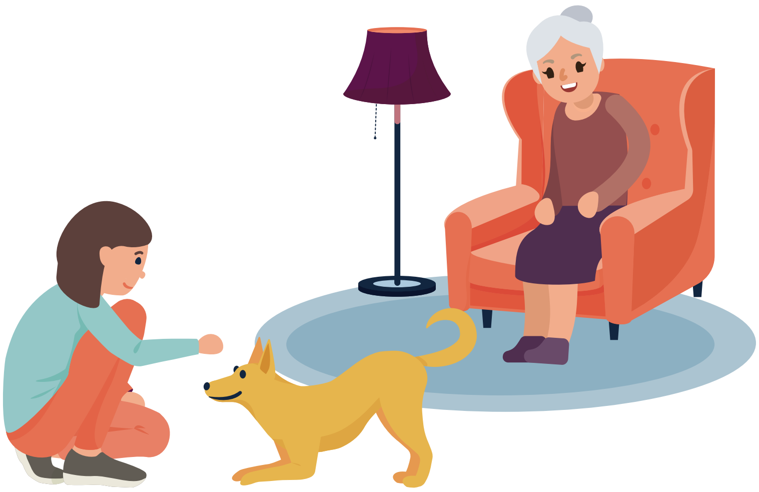

Sociaal leven

Mensen met een huisdier maken makkelijker contacten met anderen. Dieren zijn een gemakkelijk gespreksonderwerp. Ook gaat er een vorm van sociale controle van uit. Oplettende buren kunnen bijvoorbeeld gaan kijken wanneer iemand niet is gaan wandelen met de hond. Huisdieren kunnen ook voorkomen dat iemand zich eenzaam voelt. Mensen komen namelijk nooit thuis in een ‘leeg’ huis.

Interactie

Een huisdier vervult de behoefte aan emotionele nabijheid. Baasjes voelen zich veiliger wanneer het huisdier in de buurt is. Het aaien en knuffelen van een huisdier wordt vaak als fijn ervaren door zowel het baasje als het huisdier. Het baasje voelt zich hierdoor geliefd en meer ontspannen.

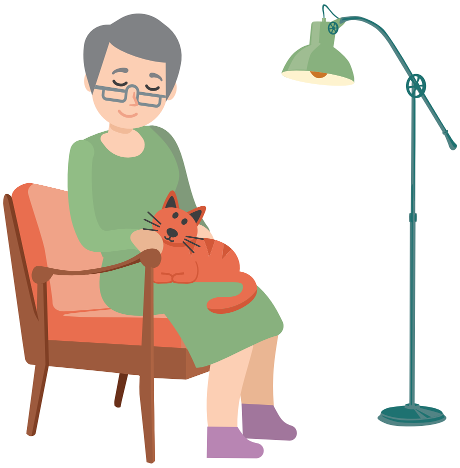

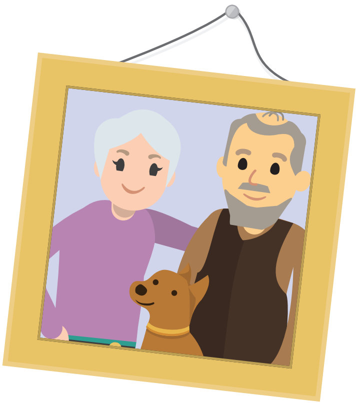

Verbinding

Baasjes ervaren vaak een sterke band met hun huisdier. Het dier wordt vaak gezien als onderdeel van het gezin. Er is wederzijdse afhankelijkheid en onvoorwaardelijke liefde. Het zorgen voor huisdieren geeft voldoening en kan zin geven aan het leven. Het huisdier zorgt ook indirect voor het baasje. Het kan emotionele steun bieden.

Herkenning en zingeving

Voor mensen die zich in een moeilijke sociale positie bevinden, kan een huisdier houvast geven. Het dier geeft invulling aan het leven van het baasje. Baasjes en huisdieren (met name honden en katten) herkennen vaak elkaars emoties. Huisdieren helpen bij het versterken van herinneringen en het geheugen.

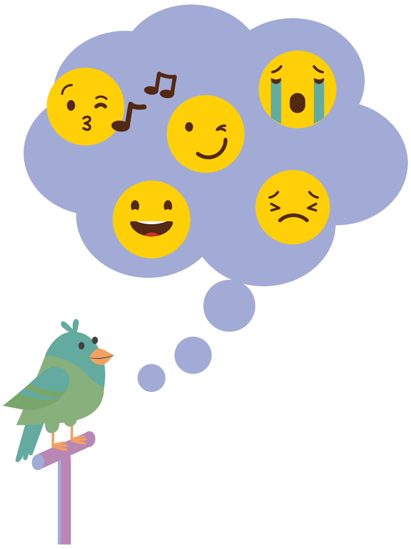

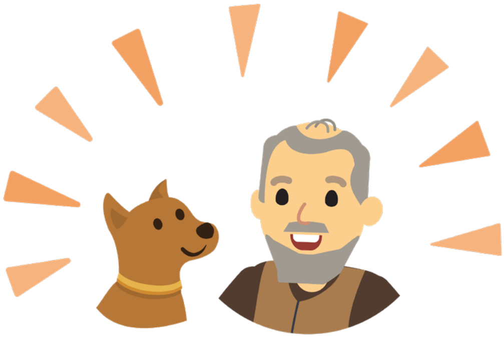

Emoties

Baasjes ervaren vaak veel emotionele steun van hun huisdier. Ze voelen zich geliefd en bovendien gesteund op sombere momenten. Andersom reageren huisdieren op de gevoelens van hun baasje. Honden kunnen bijvoorbeeld gevoelens van woede, angst of blijdschap bij hun baasje aanvoelen, en door die stemming beïnvloed worden.

Mentale gezondheid

Huisdieren dragen bij aan een betere mentale gezondheid. Het dier geeft liefde, plezier en gezelligheid. Bovendien vinden mensen het vaak fijn om ergens verantwoordelijk voor te zijn. Dan voelt men zich nuttig. Huisdieren geven daarnaast een gevoel van veiligheid en comfort. Dit heeft een positieve invloed op de geestelijke gezondheid.

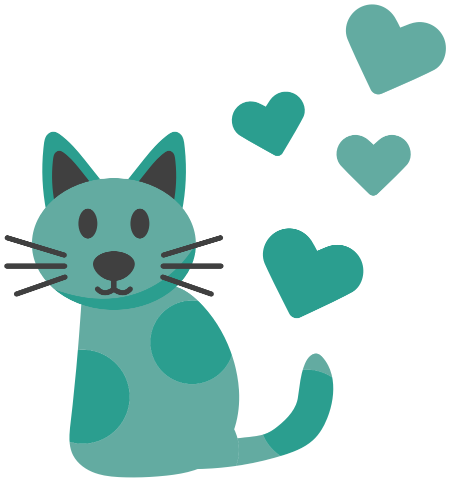

Een cliënt aan het woord:

“Ik ben graag bezig met mijn parkiet. Ik zorg graag voor hem. Eigenlijk zorgt hij op een bepaalde manier ook voor mij.”

Een mantelzorger aan het woord:

“Onze kat voelt mijn stemming precies aan. Als ik een zware dag heb gehad, komt hij even bij me liggen. Dat is heel rustgevend.”

Een cliënt aan het woord:

“Wandelen en spelen met mijn hond zorgt voor beweging. Ik voel mij daardoor fitter. Bovendien spreek ik altijd wel iemand in het park.”

4. Het welzijn van uw huisdier

Het welzijn van een huisdier straalt af op dat van het baasje, en andersom. Als het niet goed gaat met een huisdier, kan een zorgontvanger daar ook hinder van ondervinden.

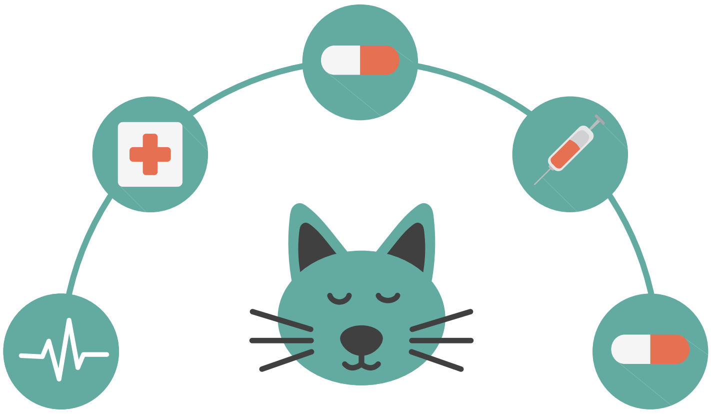

Hygiëne

In sommige gevallen kunnen huisdieren die ziek zijn ook een gevaar vormen voor de gezondheid van hun baasje. Als u thuis zorg ontvangt, is het daarom extra belangrijk te letten op bijvoorbeeld het wassen en kammen van een vacht, of het verzorgen van een wond. Schakel tijdig hulp in bij deze situaties en praat hierover met mantelzorgers en andere hulpverleners.

Een leuke omgeving voor huisdieren

Huisdieren moeten vrij zijn om zich te gedragen zoals ze dat van nature gewend zijn. Als huisdiereigenaren ziek zijn of zich niet goed kunnen bewegen, krijgen huisdieren wellicht minder aandacht en stimulatie. Verveling, stress en onwenselijk gedrag liggen dan op de loer. Ook huisdieren kunnen depressief worden.

Er is veel mogelijk om een dierenleven te verrijken. Voor huisdieren betekent verrijking dat ze activiteiten kunnen ondernemen die op natuurlijk gedrag lijken. Ze worden er ontspannen van en ervaren minder stress. Hier vindt u enkele voorbeelden van omgevingsverrijking voor honden en katten van zorgontvangers.

Speelgoed voor huisdieren

Denk aan een bal, kauwspeeltjes of zelfs een speelhuis voor sommige types huisdieren.

Ballenmachine

Leuk vermaak voor de hond, binnenshuis of in de tuin.

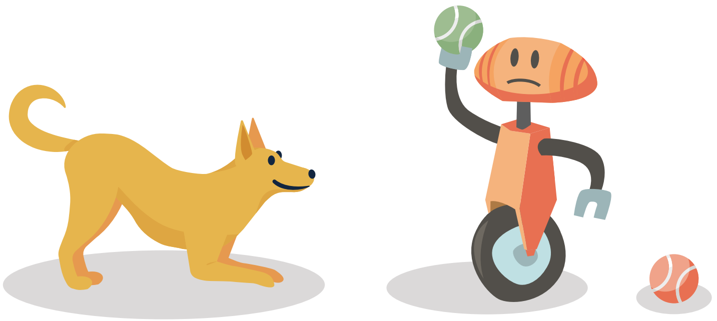

Voerpuzzel

Het zoeken naar voedsel is natuurlijk gedrag voor veel dieren. Een voerpuzzel is een extra uitdaging voor binnenshuis.

Likmat

Een likmat geeft extra uitdaging en lijkt op natuurlijk gedrag.

Uitlaatservice

Contact met andere dieren is belangrijk voor het geestelijke en lichamelijke welzijn van huisdieren. Naast afspraken hierover met naasten of buren kan een uitlaatservice hiervoor een oplossing zijn. Deze laatste kost in de meeste gevallen geld.

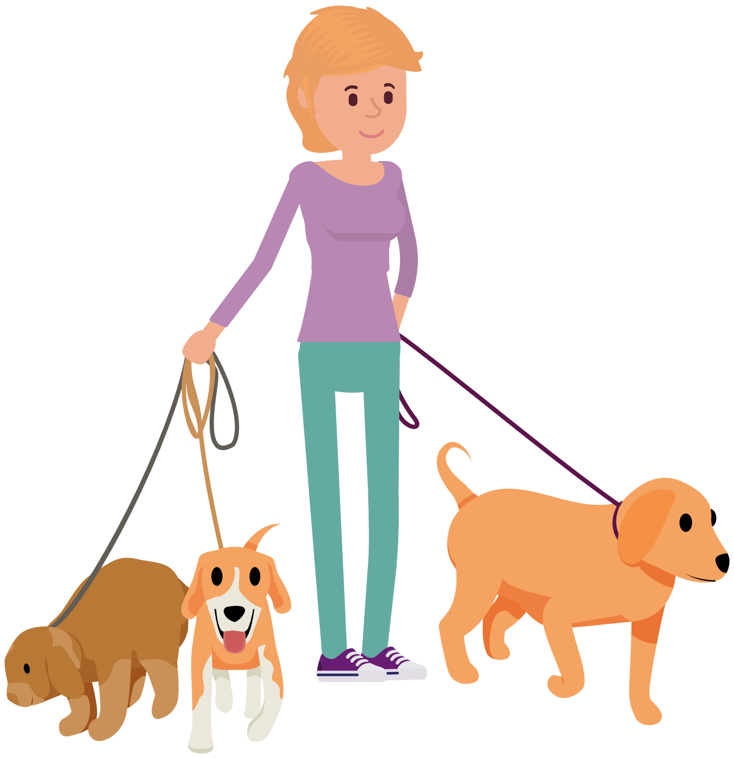

5. Ondersteuningsmogelijkheden

Soms is het nodig om extra ondersteuning te zoeken in de zorg voor uw huisdier(en). Denk hierbij aan familie, vrienden, buren of vrijwilligers. Ook zijn er organisaties waar u (eventueel tijdelijk) op kunt leunen en technische oplossingen die u bij de zorg van uw huisdier kunnen helpen. Extra handen bij de zorg voor uw huisdier dus! Het is belangrijk u hier tijdig op te oriënteren en afspraken vast te leggen. Volg de stappen hieronder om u hierbij te helpen.

1. Hulpmiddelen

Zijn er hulpmiddelen beschikbaar die het u makkelijker maken om voor uw huisdier te zorgen? Denk aan praktische en technische oplossingen (zie volgende pagina).

2. Hulp van naasten

Heeft u mensen in uw naaste omgeving die u kunnen ondersteunen? Maak afspraken met hen en leg deze vast.

3. Hulp van anderen

Hoe kunnen buren of (betaalde) vrijwilligers u ondersteunen? Maak afspraken en leg deze vast.

4. Hulp van externe organisaties

Er zijn veel organisaties die u kunnen ondersteunen. Denk bijvoorbeeld aan een dierenvoedselbank, een minimadierenarts of de Dierenbescherming.

Automatisering

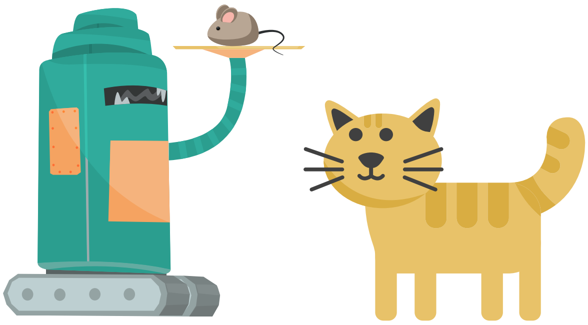

Sommige zorgtaken voor huisdieren kunnen worden geautomatiseerd. Een automatische voederbak is hier een voorbeeld van, of een kattenbak die zichzelf automatisch reinigt. Een GPS-tracker kan bijvoorbeeld helpen om het huisdier makkelijker terug te vinden als het aan uw aandacht is ontsnapt. Technische oplossingen zoals sociale robots of een dementieklok kunnen u aan zorgtaken voor uw huisdier helpen herinneren.

Geautomatiseerde oplossingen moeten natuurlijk aangeschaft worden en hebben onderhoud nodig. Zo moet bijvoorbeeld een voederbak bijgevuld of schoongemaakt worden. Overleg met uw naasten of u deze ondersteuning nodig heeft en hoe u dit kunt realiseren.

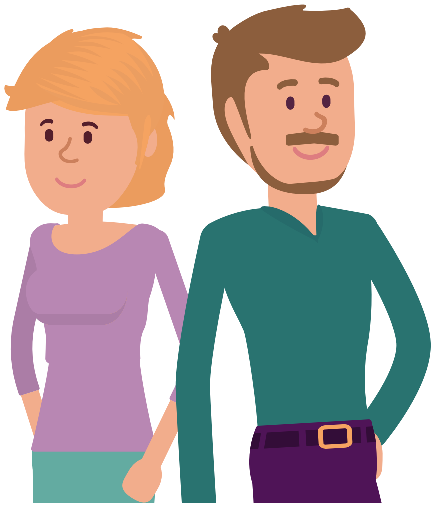

Vrijwilligers

Als familieleden, vrienden of buren niet kunnen helpen bij het zorgen voor uw huisdier, kunt u vrijwilligers benaderen. Er zijn diverse initiatieven zoals het Maatjes project, de Dierenbuddy (van de Dierenbescherming) en websites voor een uitlaatservice of andere vrijwillige hulp. Ook bestaan er (telefoon)apps zoals Nextdoor, waar een hulpvraag op geplaatst kan worden. Eventueel kunnen wijk- en dorpsondersteuners helpen bij het zoeken naar vrijwilligers.

Ondersteuning in de kosten

Personen met een beperkt budget kunnen in aanmerking komen voor (financiële) steun bij de aanschaf van dierenvoeding of bij het dragen van medische kosten voor hun huisdier. In sommige gevallen is het mogelijk dat hiervoor inzicht in uw financiën gevraagd wordt.

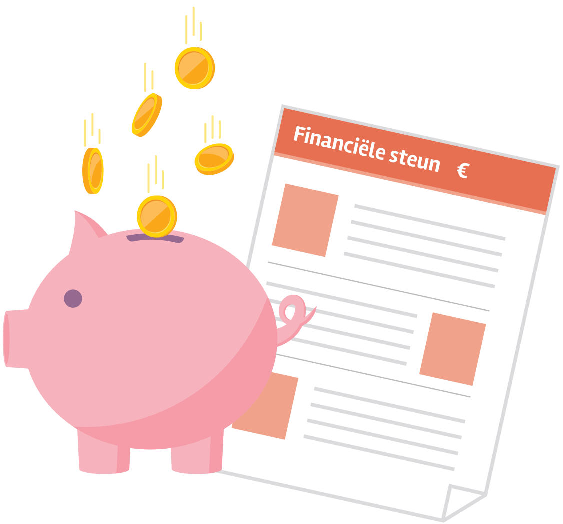

Hieronder staan enkele voorbeelden van zaken en taken waarover u met uw familie en vrienden kunt praten. Schrijf de gemaakte afspraken op.

Eten en drinken:

- Wie geeft het huisdier eten en drinken?
- Waar staat de voerbak, de waterbak en het voer?
- Hoeveel, hoe vaak en hoe laat krijgt het huisdier eten? (natvoer of brokken?)

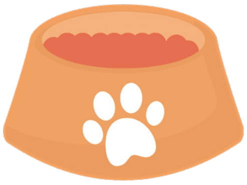

Zichzelf kunnen zijn:

- Wie laat het huisdier uit, hoe vaak en hoe laat?
- Heeft het dier een eigen plek waar het zich kan terugtrekken? Waar is dat?
- Wordt het dier altijd aan de lijn gehouden, of soms ook losgelaten?
- Hoe gedraagt het dier zich tegenover andere dieren en mensen?

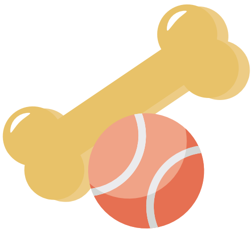

De juiste zorg krijgen:

- Wie maakt de plek van het huisdier schoon?
- Heeft het dier een paspoort en een chip en is het gekoppeld aan de huidige eigenaar?
- Hoe vaak en hoe moet het dier worden verzorgd?

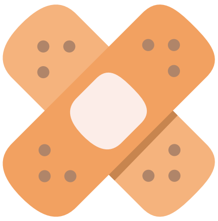

Gezondheid:

- Wie gaat met het huisdier naar de dierenarts?
- Gebruikt het dier medicijnen?
- Welke gezondheidsproblemen heeft het dier en moet daar rekening mee worden gehouden?

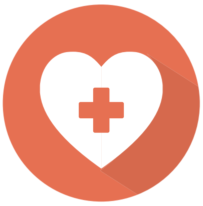

Gelukkig zijn:

- Wat maakt het dier blij?
- Wat is het karakter van het dier bijvoorbeeld verlegen of terughoudend?
- Vertoont het dier wel eens agressief gedrag?

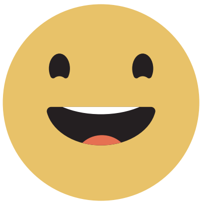

6. PET@home checklist

Afspraken voor huisdierbezitters
en hun naasten

PET@home
Als het baasje zorg nodig heeft

Datum: ______________________________

Soort huisdier en naam: ______________________________

Geboortedatum huisdier: ______________________________

Eigenaar: ______________________________ **Telefoon:** ________________

Tweede contactpersoon: ______________________________ **Telefoon:** ________________

Dierenarts: ______________________________

Dierenambulance: 144

Chipnummer huisdier:

____

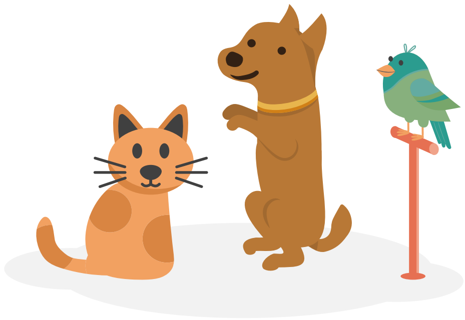

Karakter/algemene indruk/bijzonderheden van het huisdier:

(denk aan gezondheid of gedrag van het huisdier tegen onbekenden, het lievelingseten of vaste tijden waarop het dier wordt uitgelaten):

____

____

____

____

____

____

____

____

____

____

____

____

____

____

Zijn er afspraken gemaakt over het huisdier? En zo ja, welke en met wie?

Toelichting:

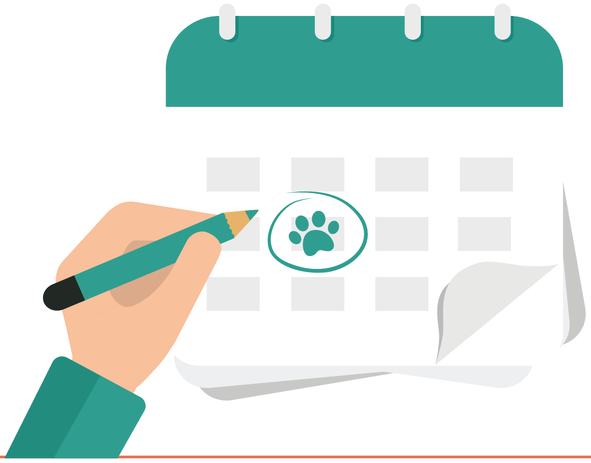

7. Afscheid

Het kan moeilijk zijn om afscheid te moeten nemen van een huisdier. Hier staan enkele tips die u kunnen helpen wanneer een huisdier moet worden herplaatst omdat u er zelf niet meer voor kunt zorgen, of wanneer een huisdier overlijdt.

Herplaatsing van een huisdier

Wanneer uw huisdier herplaatst moet worden en dit niet lukt bij bekenden, moet een huisdier ergens anders ondergebracht worden. Hiervoor kan een nieuw baasje op internet worden gezocht, of er kan een (lokaal) dierenasiel worden benaderd.

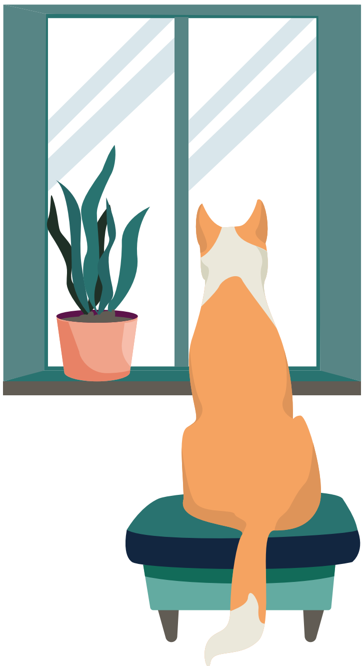

Neem uw tijd om een goed adres te vinden voor uw huisdier. Oudere dieren die gewend zijn aan een rustige omgeving zullen het waarschijnlijk niet goed doen op een drukkere plek.

Daarnaast kan het prettig zijn voor het dier als zijn gewoontes en lievelingseten bekend zijn bij de nieuwe eigenaar. Dat maakt de overstap wellicht makkelijker.

Zorg voor uzelf dat er een goed afscheid plaatsvindt en dat u uw huisdier met een gerust hart kunt overdragen.

Overlijden van een huisdier

Het verlies van een huisdier heeft vaak een groot effect op het baasje. Met name bij ouderen kan het gemis sneller tot eenzaamheid leiden. Vaak is het door de leeftijd niet altijd meer mogelijk om een nieuw huisdier aan te schaffen. De sterkte van de emoties die vrijkomen bij de dood van een huisdier, hangt af van de band die u heeft met het huisdier.

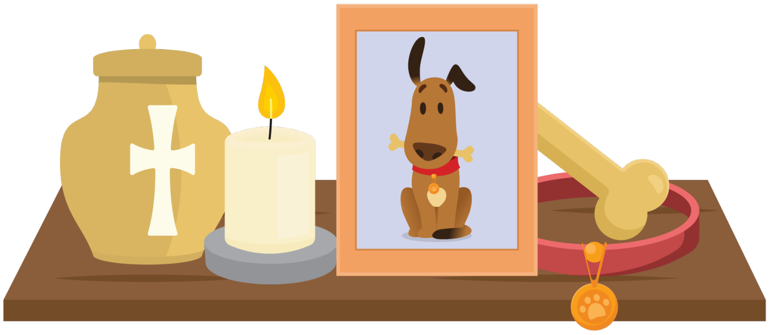

Misschien wilt u een begrafenis of crematie voor uw huisdier organiseren. Wellicht wilt u foto's van uw huisdier in huis ophangen, of de urn met de as van uw huisdier bewaren. Dit is voor iedereen anders. Het is belangrijk om met naasten te praten over het verlies en de emoties die u ervaart.

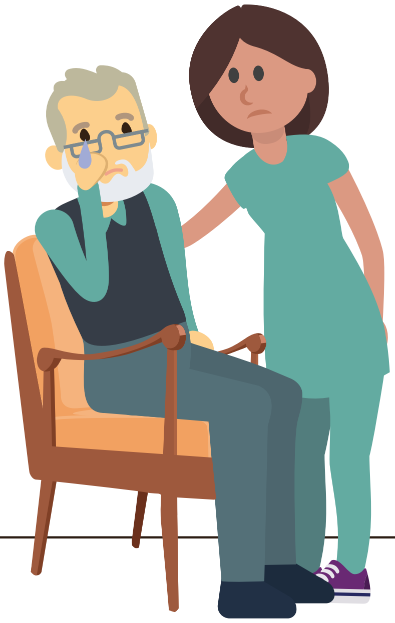

U kunt ook uw zorgmedewerker deelgenoot maken van de gebeurtenis en vertellen hoe u zich voelt. Voor de zorgverlener is het namelijk van belang dat hij/zij weet hoe het met u gaat. Bovendien kunnen deze gesprekken ook troost bieden en helpen bij het rouwproces.

8. Een nieuw huisdier

Een huisdier is een hele verantwoordelijkheid, maar heeft ook veel voordelen. Overweegt u het (opnieuw) nemen van een huisdier? Onderstaande informatie kan u helpen bij het maken van een beslissing.

Wel of geen huisdier?

Als u over een nieuw huisdier nadenkt, zijn er veel zaken te overwegen. Zo zijn er kosten aan verbonden en moet er aan de behoeftes van een huisdier worden voldaan, bijvoorbeeld beweging buitenshuis of verschoning van een kooi of bak. Soms is het hebben van een (nieuw) huisdier juist een goed idee, vanwege de voordelen die een huisdier met zich meebrengt (zie Hoofdstuk 4).

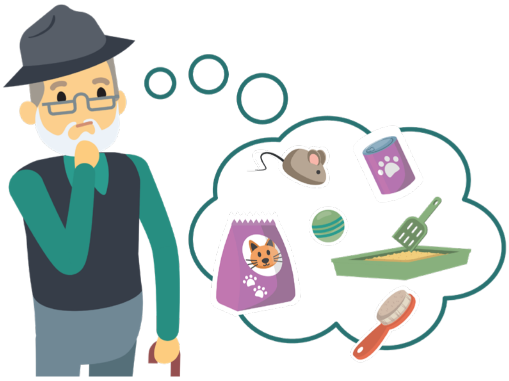

Om in te schatten welke gevolgen uw keuze op uw dagelijkse leven heeft, kunt u over uw huisdierwens praten met uw naasten en uw mantelzorgers. Als u de voor- en nadelen goed afweegt, is de kans groot dat u en uw eventuele huisdier gelukkig met elkaar worden.

Geschikte dieren

Voor oudere of zorgbehoevende personen zijn oudere dieren vaak geschikt. Ze zijn meestal rustiger en hebben minder behoefte aan beweging. Een ouder dier vindt u bijvoorbeeld in een asiel. Mocht u dit overwegen, vraag dan goed na of het dier zindelijk en gesocialiseerd is. Indien u een (zwerf)dier uit het buitenland wilt halen, wees dan extra oplettend. Dieren met trauma of gedrags- of gezondheidsproblemen zullen veel aandacht van u vragen.

Alternatieven voor een huisdier

Als u niet in staat bent om zelf voor een huisdier te zorgen, maar u vindt het wel fijn om structureel contact met dieren te hebben, dan kunt u overwegen om dierenoppas te worden. U kunt hiermee uw eigen leven verrijken en tegelijkertijd iemand anders helpen.

Voor mensen die behoefte hebben aan sociaal contact, maar niet de mogelijkheid hebben om voor een huisdier te zorgen, kan een robotdier een oplossing zijn. Uit diverse onderzoeken blijkt dat een robothuisdier een positieve invloed heeft op met name mensen met dementie en aandoeningen in het autisme spectrum.

Checklist voor het aanschaffen van een huisdier

- Bespreek tijdig met uw mantelzorgers de eventuele gevolgen van uw huisdier voor hen.
- Maak een overzicht van benodigde spullen en de kosten hiervan (bijvoorbeeld speelgoed en voer).
- Onderzoek tijdig of naasten, vrienden of buren kunnen ondersteunen bij zorgtaken voor het huisdier, mocht dit onverhoopt nodig zijn.
- Zoek een dierenarts in de buurt.
- Onderzoek of het nodig is om het huisdier te chippen en te registreren via www.chipjedier.nl.
- Overweeg een verzekering af te sluiten voor het huisdier.
- Laat de buren weten dat er een huisdier op komst is zodat het geen verrassing is.
- Maak extra tijd vrij voor de eerste weken dat het dier in huis komt. Het moet wennen en heeft extra aandacht nodig.

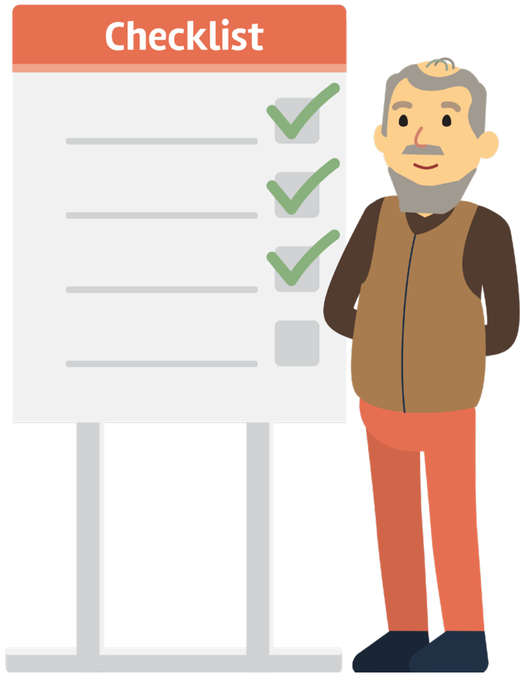

9. Handige websites en zoektips

Hier vindt u een overzicht van nuttige websites en enkele tips voor het zoeken naar informatie voor u en uw huisdier.

Website tips

Algemene informatie over huisdieren

www.licg.nl (Landelijk Informatiecentrum Gezelschapsdieren)

Ondersteuning bij het houden van huisdieren

www.dierenartskiezen.nl

www.dierenbescherming.nl/preventie (Telefoonnummer: 088 8113 000)

www.nlvoorelkaar.nl

Informatie over huisdierenoppas

www.oopoeh.nl

www.hondjeuitlaten.nl

Informatie over hygiëne en ziektes

www.rivm.nl/ziek-door-dier/besmettingsroutes/huisdieren (RIVM)

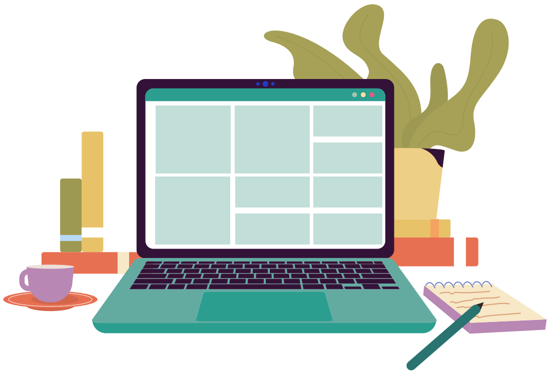

Zoektips

Op zoek naar vrijwilligers?

Gebruik de zoektermen 'vrijwilligers', 'huisdieren', en de naam van uw woonplaats

Op zoek naar inspiratie over omgevingsverrijking?

Kijk op Youtube (www.youtube.com) en gebruik daar de zoektermen 'verrijking hond' of 'verrijking …' (vul uw type huisdier in)

Op zoek naar geautomatiseerde oplossingen?

Gebruik de zoektermen ‘huisdieren’ en ‘automatisering’ of ‘domotica’

Op zoek naar een robothuisdier?

Gebruik de zoektermen ‘robotdier’ of ‘robothuisdier’ en eventueel ‘bestellen’

Op zoek naar (tijdelijke) herplaatsing van huisdieren?

Gebruik de zoektermen ‘huisdier’, ‘herplaatsen’ en eventueel ‘tijdelijk’

Op zoek naar financiële ondersteuning bij het houden van uw huisdier?

Gebruik de zoektermen ‘noodfonds’ of ‘dierenvoedselbank’ of ‘minimadierenarts’ samen met de naam van uw woonplaats

Noteer hieronder lokale ondersteuningsmogelijkheden met telefoonnummers:

Afspraken poster

PET@home
Als het baasje zorg nodig heeft

Geef aan wie u helpt, waarmee en hoe ze bereikt kunnen worden.

Hang deze poster op een plek waar u hem goed kunt zien.

Cliënt/eigenaar

Contactpersoon huisdier

Sociaal netwerk

Contactpersoon zorg

Dierenarts

Eten en drinken

Zichzelf kunnen zijn

De juiste zorg

Gezondheid

Gelukkig zijn

ZonMw Open Universiteit Radboudumc UKON Universitair Kennisnetwerk Ouderenzorg Nijmegen de Zorgboog voor alle generaties

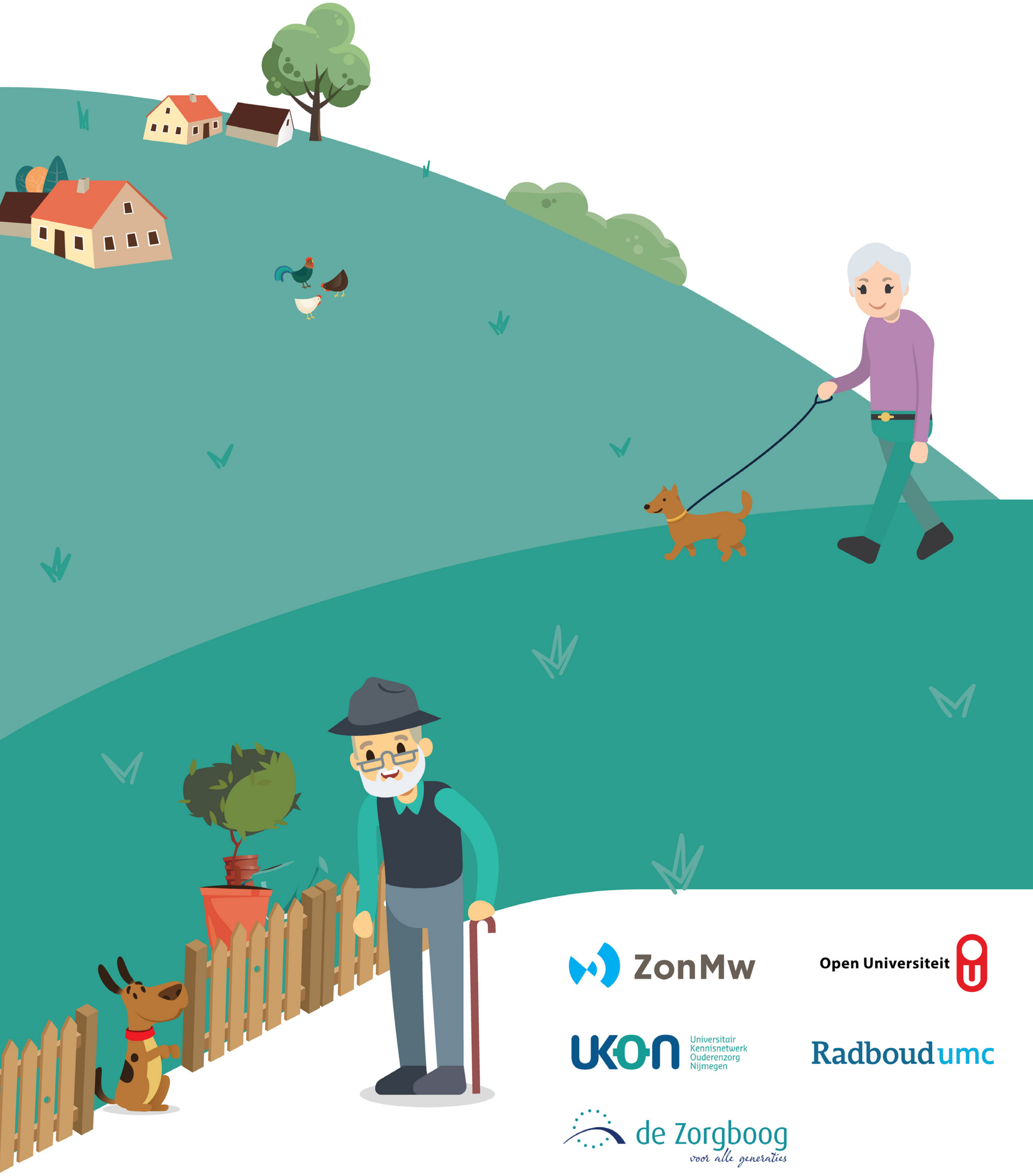

ZonMw

Open Universiteit

UKON Universitair Kennisnetwerk Ouderenzorg Nijmegen

Radboudumc

de Zorgboog voor alle generaties
