# Supplementary material for: The development of the PET@home toolkit: An experience-based co-design method study
Source: Int J Nurs Stud Adv. 2024 Mar 6;6:100189. doi: 10.1016/j.ijnsa.2024.100189 (PMC11080344; doi:10.1016/j.ijnsa.2024.100189)
Supplement: Supplementary file 6 [file mmc6.pdf]

Dierenwelzijn

Voor zorgmedewerkers

PET@home
Als het baasje zorg nodig heeft

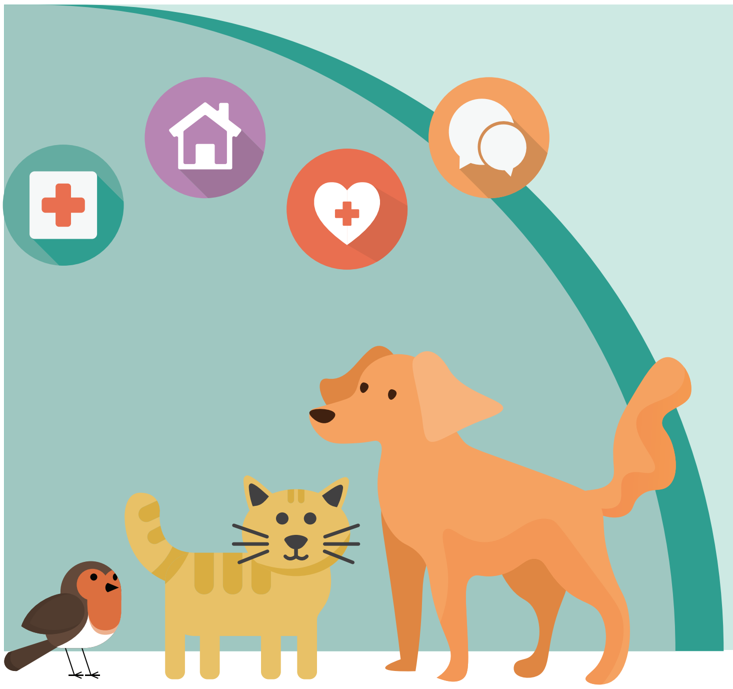

Het is belangrijk dat er aandacht is voor het welzijn van het huisdier, zowel vanuit de zorgontvanger als vanuit de zorgmedewerker.

Behoeftes en welzijn huisdieren

Er zijn veel verschillende soorten huisdieren. Voor informatie over een grote variëteit aan huisdieren kan onder andere de website van het Landelijk Informatiecentrum Gezelschapsdieren (www.licg.nl) geraadpleegd worden.

Om te constateren of in een thuissituatie aan dierenwelzijn wordt voldaan, zijn de ‘vijf vrijheden voor (huis)dieren’ een goede leidraad:

1. Dieren zijn vrij van dorst, honger en onjuiste voeding
2. Dieren zijn vrij van fysiek en thermaal (te warm of te koud) ongerief
3. Dieren zijn vrij van pijn, verwonding en ziektes
4. Dieren zijn vrij van angst en chronische stress
5. Dieren zijn vrij om hun natuurlijk gedrag te vertonen; denk hierbij aan dat het huisdier vrij moet zijn om te kunnen spelen en ‘zichzelf’ kan zijn.

ZonMw · Open Universiteit · Radboudumc · UKON Universitair Kennisnetwerk Ouderenzorg Nijmegen · de Zorgboog voor alle generaties

Dierenwelzijn
Voor zorgmedewerkers

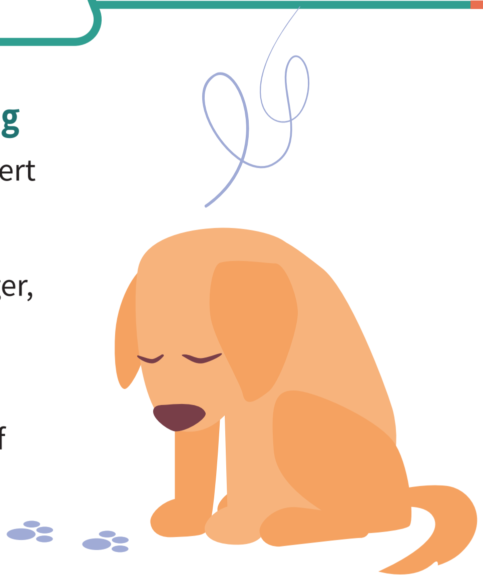

Vermoeden van dierenverwaarlozing of -mishandeling

Wanneer de aandacht voor het welzijn van het huisdier verandert bij een zorgontvanger, kan dit een indicatie zijn dat er iets (medisch of anderszins) aan de hand is met de zorgontvanger. Als u dit signaleert, bespreek dit dan met de cliënt, mantelzorger, contactpersoon of zorgverantwoordelijke. Het kan onbedoeld zijn en een gevolg van iets anders dat speelt.

Heeft u vermoedens van opzettelijke dierenmishandeling en/of verwaarlozing, spreek dan de eigenaar van het huisdier of de contactpersoon voor het huisdier aan of meld dit bij de zorgverantwoordelijke.

Dierenbescherming

De Dierenbescherming heeft materiaal ontwikkeld wat kan helpen bij het constateren van mogelijke problemen. Zo is er een “Signalenkaart van dierenmishandeling verwaarlozing” en een “Afwegingskader Bepalen van ernst van de situatie bij dierenverwaarlozing en mishandeling”.

Deze materialen zijn te verkrijgen via de website van de Dierenbescherming (via Preventieve dierenhulp) en via de Dierenbescherming helpdesk hulpverleners, telefoonnummer **088-8113000**. De helpdesk kan ook worden benaderd voor overleg en advies wanneer zorgverleners worden geconfronteerd met verstoord dierenwelzijn.

Huiselijk geweld

Indien er sprake is van dierenmishandeling, is er een verhoogd risico op huiselijk geweld. Wees hier alert op! Wanneer iemand hulp wil voor huiselijk geweld maar het huisdier niet wil achterlaten bij de agressor kan contact opgenomen worden met stichting Mendoo (https://mendoo.nl/)

Wetgeving

Met betrekking tot dierenmishandeling zijn bepalingen opgenomen in de Wet Dieren artikel 2.1.

In nood

In noodgevallen kan contact opgenomen worden met Meldpunt 144.

Bel hiervoor 144.

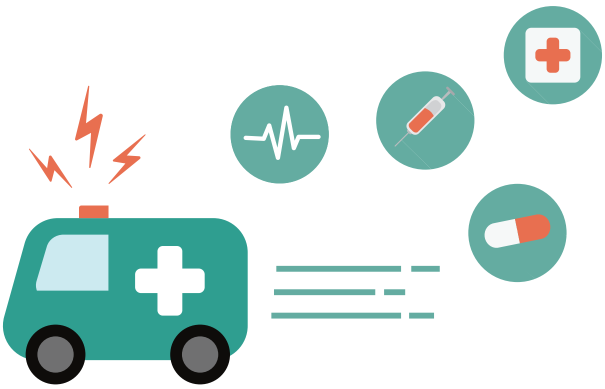

Mede mogelijk gemaakt door:

ZonMw · Open Universiteit · Radboudumc · UKON Universitair Kennisnetwerk Ouderenzorg Nijmegen · de Zorgboog voor alle generaties
