# Supplementary material for: The development of the PET@home toolkit: An experience-based co-design method study
Source: Int J Nurs Stud Adv. 2024 Mar 6;6:100189. doi: 10.1016/j.ijnsa.2024.100189 (PMC11080344; doi:10.1016/j.ijnsa.2024.100189)
Supplement: Supplementary file 7 [file mmc7.pdf]

Communicatietips

Voor zorgmedewerkers

PET@home
Als het baasje zorg nodig heeft

Huisdier als gespreksonderwerp

Mensen vinden het fijn om over hun huisdier te praten. Gebruik het huisdier als een laagdrempelig gespreksonderwerp. Een gesprekje over het huisdier kan het ijs breken.

Tip: Gebruik de gesprekskaarten ter inspiratie.

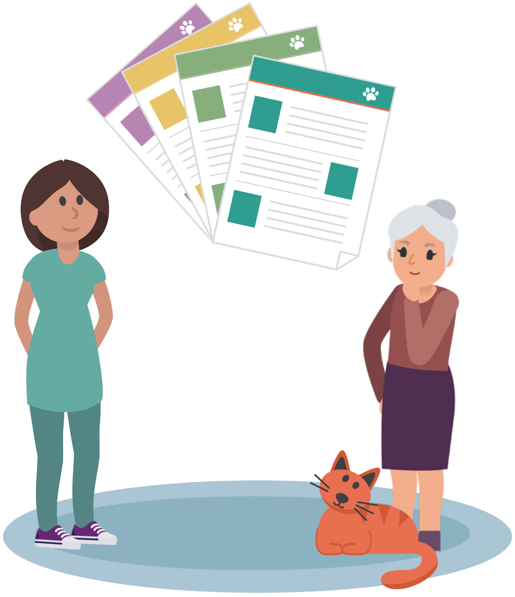

Erken de zorg voor het huisdier

Zorgen voor een huisdier vraagt veel, zeker bij personen die zelf hulp ontvangen en hun naasten. Benoem deze zorg en geef complimenten. Dit biedt ook ruimte aan de cliënt om aan te geven wat steeds minder goed gaat en waar in de toekomst wellicht hulp bij nodig is.

Tip: Zorg ervoor dat complimenten oprecht zijn. Bedenk eerst wat de zorg voor het huisdier voor de cliënt betekent.

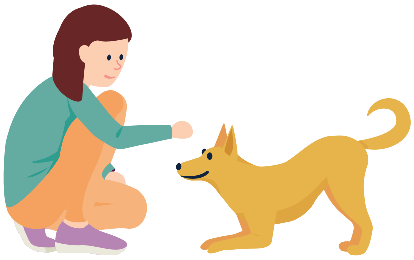

Aandacht voor het huisdier

Een huisdier maakt deel uit van iemands leven of gezin. Schenk daarom ook aandacht aan het huisdier. Leef mee en zoek eventueel interactie op.

Tip: Neem hierbij de nodige hygiëne in acht. Was uw handen na fysiek contact met het huisdier.

Oordeel niet over de geboden zorg

Normen van uw cliënten kunnen afwijken van uw eigen normen. Als u vermoedt dat de zorg niet optimaal verloopt, oordeel dan niet, maar ga het gesprek aan.

Tip: Vraag wat men zelf vindt en waar men hulp bij nodig heeft. Geef ook aan waar men hulp bij kan krijgen. Verwijs naar de PET@home brochure. Raadpleeg eventueel ook de leaflet Dierenwelzijn.

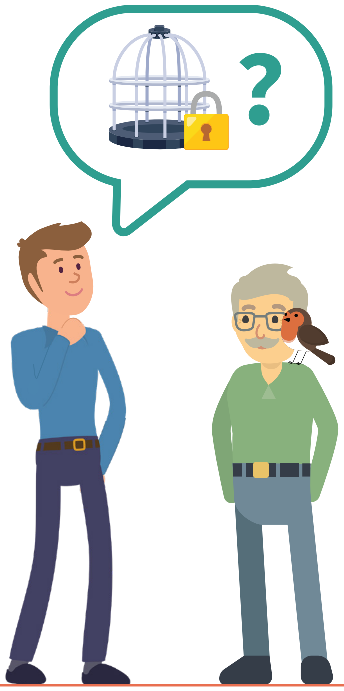

Bied ondersteuning

Stimuleer de cliënt om voor het huisdier te zorgen. Denk samen in termen van oplossingen en probeer concrete afspraken te maken.

Tip: Gebruik voor ideeën en afspraken de PET@home brochure. Laat een 'afsprakenposter' of 'afsprakenchecklist' achter bij de cliënt.

Schep heldere verwachtingen

Cliënten en naasten zijn zelf verantwoordelijk voor de zorg voor het huisdier. Wees vanaf het begin helder over verwachtingen over en weer met betrekking tot het huisdier. Bespreek bijvoorbeeld met de cliënt en diens naasten wanneer u het prettig vindt dat het huisdier apart wordt gezet tijdens zorgtaken.

Tip: Zijn er afspraken binnen de zorgorganisatie? Bespreek dit met collega's en trek één lijn.

ZonMw | Open Universiteit | Radboudumc | UKON Universitair Kennisnetwerk Ouderenzorg Nijmegen | de Zorgboog
