# Supplementary material for: The development of the PET@home toolkit: An experience-based co-design method study
Source: Int J Nurs Stud Adv. 2024 Mar 6;6:100189. doi: 10.1016/j.ijnsa.2024.100189 (PMC11080344; doi:10.1016/j.ijnsa.2024.100189)
Supplement: Supplementary file 8 [file mmc8.pdf]

Praktische tips voor zorgorganisaties

- Stel één contactpersoon aan met betrekking tot issues rondom dierenwelzijn
- Stel duidelijke regels en procedures op:
  - Over waar de verantwoordelijkheid voor het huisdier ligt
  - Over hoe omgegaan moet worden met huisdieren
  - Voor het delen van informatie over de huisdieren
- Draag aan medewerkers uit dat huisdieren een onderdeel zijn van het systeem van zorgcliënten en belangrijk zijn
- Laat medewerkers onderling afspraken maken over huisdieren van cliënten waar ze zorg verlenen
- Leg afspraken tussen cliënten, hun naasten en de zorgorganisatie/medewerkers vast in een huisdierenovereenkomst of zorgleefplan
- Voeg huisdieren toe als onderwerp in de door de organisatie gebruikte zorgplansystematiek. Noteer hierin bijvoorbeeld de aanwezigheid van het huisdier, de contactgegevens van een tweede contactpersoon, contactgegevens van een (tijdelijk) opvangadres en andere bijzonderheden zoals het gedrag van het huisdier naar vreemden
- Voorzie in supervisie/intervisie momenten voor het delen van ervaringen/good practices
- Breng cliënten met huisdieren samen voor het delen van tips (bijvoorbeeld tijdens een jaarlijkse bijeenkomst)
- Benader de Helpdesk Hulpverleners van de Dierenbescherming als advies nodig is
  https://www.dierenbescherming.nl/werkzaamheden/preventie/helpdesk-hulpverleners

  Hier kunnen ook actuele overzichten worden opgevraagd van:

  - Ondersteuning aan huis
  - Bemiddelingswebsites voor herplaatsing
  - Neutralisatieacties bij dierenartsen
  - Dierenvoedselbanken
  - Fondsen voor (on)verwachte medische kosten
  - Gemeentelijke regelingen voor minima
  - Minimadierenartsen
  - Noodopvang van huisdieren

- Leg een document aan met lokale initiatieven met betrekking tot bovenstaande overzichten. Voeg deze met telefoonnummers toe als inlegvel aan de PET@home informatiebrochure en maak het bestand vindbaar voor medewerkers
- Raadpleeg www.licg.nl, de website van het Landelijk Informatiecentrum voor Gezelschapsdieren voor informatie over een grote variëteit aan huisdieren
